# Cauda Equina Syndrome: A Survey of Guideline Utilisation in Primary Care in England

**DOI:** 10.1002/msc.70129

**Published:** 2025-06-05

**Authors:** Jonathon Gill, Sue Greenhalgh, Jos M. Latour, Gillian Yeowell

**Affiliations:** ^1^ Spinal Surgery Service Somerset NHS Foundation Trust Taunton UK; ^2^ School of Health Professions Faculty of Health, University of Plymouth Plymouth UK; ^3^ Orthopaedic Interface Service Bolton NHS Foundation Trust Bolton UK; ^4^ Department of Health Professions Faculty of Health and Education Manchester Metropolitan University Manchester UK; ^5^ School of Nursing Faculty of Health University of Plymouth Plymouth UK; ^6^ South West Clinical School Somerset NHS Foundation Trust Taunton UK

**Keywords:** cauda equina syndrome, CES, clinical practice guideline, getting it right first time (GIRFT), knowledge mobilisation, primary care

## Abstract

**Background:**

Cauda equina syndrome (CES) is a spinal emergency. Over half of known cases first present to primary care for initial assessment. In February 2023, the Getting It Right First Time (GIRFT) national programme launched new CES guidelines, which included an important change in practice: a new urgent referral route.

**Aim:**

This study aims to explore the awareness and use of the GIRFT guidelines in a primary care setting in England.

**Design and Setting:**

A cross‐sectional online survey was used to collect data from primary care clinicians working across England.

**Method:**

Using purposive sampling, the survey was shared with primary care clinicians across England and conducted between 21‐10‐2024 and 24‐12‐2024. The RE‐AIM framework underpinned the survey design. Descriptive analysis was employed to interpret frequency and Likert data.

**Results:**

A total of 515 responses were received from across all 42 integrated care boards in England. Of the 515 participants, 452 (88%) were aware of a CES guideline or pathway, with 297/515 (58%) being aware of the GIRFT guidelines. Two‐thirds had access to a local CES pathway (*n* = 304/452, 67%). Nearly all clinicians highlighted that consulting either a local CES pathway or national guidelines supported their clinical decision making.

**Conclusion:**

This is the first study to investigate the awareness and utilisation of the GIRFT guidelines in primary care across England. The use of locally agreed CES pathways was shown to increase adherence to their recommendations in primary care. These findings suggest that using up‐to‐date local CES pathways can increase adherence to the GIRFT guidelines.

## 1 Introduction

In the United Kingdom, over half of patients with confirmed CES first present to a primary care clinician for assessment (Woodfield et al. 2023). Although rare, CES is a devastating spinal emergency caused by compression of the lumbosacral nerve roots in the lumbar spine canal (Hazelwood et al. 2019; Hoeritzauer et al. 2020; Korse et al. 2017). A diagnosis of CES is only confirmed with the presence of both the clinical features of CES (Box 1) and the evidence of cauda equina compression on a magnetic resonance imaging (MRI) scan (Greenhalgh et al. 2018; Hussain et al. 2018). Long‐term negative outcomes are common in patients who experience CES, resulting in physical, psychological, and financial sequelae (Barker et al. 2021), with 21% of patients not returning to employment 1‐year post surgery (Woodfield et al. 2023). In addition, when compared to the incidence rate (Hoeritzauer et al. 2020), CES holds a disproportionally high medicolegal profile (Hutton 2019) that costs the National Health Service (NHS) over £18m per year (NHS Resolution 2020). Both of these factors can be improved by timely diagnosis.

**BOX 1 Features required for the diagnosis of cauda equina syndrome.**

1. **Clinical features**
  Spinal or lower limb radicular pain in association with:
  - Bladder dysfunction, or
  - Bowel dysfunction, or
  - Sexual dysfunction, or
  - Saddle sensory changes
2. **Radiological features**
  - Cauda equina compression on a magnetic resonance imaging scan

In February 2023, the Getting It Right First Time (GIRFT) national programme (Getting It Right First Time (GIRFT) 2025) launched a ‘National Suspected Cauda Equina Syndrome (CES) Pathway’ (Getting It Right First Time (GIRFT) 2023), which included a new urgent referral route. For clarity in this paper, these guidelines will be referred to as the *GIRFT guidelines*. The GIRFT guidelines introduced an important change in the management of patients with suspected CES during their first consultation. Patients are now categorised into either an emergency or urgent referral route, whereas previously, they were only directed through an emergency route (Germon et al. 2015). Emergency referrals require same day assessment and MRI scan, commonly via the Emergency Department or on‐call orthopaedic/neurosurgery spinal services. The new urgent referral route indicates that patients with stable CES symptoms for over 14 days (Box 1), or with isolated bilateral lower limb radicular pain, could instead be referred to a musculoskeletal (MSK) triage service for an urgent assessment within 2 weeks.

Currently, no research describes the success in translating the GIRFT guidelines into primary care. Previous studies have shown that adherence to spinal clinical practice guidelines is suboptimal (Fourney et al. 2011); this includes adherence to CES guidelines in a secondary care setting (Fountain et al. 2019; Higginson et al. 2020). To improve patient care, the GIRFT guidelines need widescale implementation across the NHS. This level of implementation is complex and time consuming (Hull et al. 2019), with the translation of evidence into clinical practice unpredictable, and if successful, taking many years (Balas and Boren 2000; Glover et al. 2018; Tabak et al. 2012). Clinical pathways are one suggested approach for supporting the translation of evidence into practice (Allen et al. 2009; Lawal et al. 2016); however, effective implementation of clinical pathways is paramount to maximise impact (Jabbour et al. 2013). With 56% of patients with confirmed CES first presenting to primary care (Woodfield et al. 2023), the implementation of the GIRFT guidelines into this setting is a vital first step. In particular, inadequate implementation could result in a delayed diagnosis and potentially long‐term negative patient outcomes.

The aim of this study is to explore the awareness and utilisation of the GIRFT guidelines for suspected CES in healthcare professionals working in a primary care setting in England. The study will also describe the translation of the GIRFT guidelines into clinical pathways in primary care.

## 2 Methods

### 2.1 Ethics

Ethical approval was obtained from the Health Research Authority (Ref: 24/HRA/3406).

### 2.2 Design

A cross‐sectional, online survey, design was employed, and reported utilising the Checklist for Reporting Results of Internet E‐Surveys ‘CHERRIES’ reporting guidelines (Eysenbach 2004). The initial concept for the survey was developed following a patient and public involvement and engagement (PPIE) consultation with patients who had lived experience of a CES referral pathway, and primary care clinicians' accounts of their varied access to CES referral pathways.

### 2.3 Sample/Recruitment Process

A purposive sample was obtained from registered healthcare professionals who assess and/or treat patients with spinal pain and work in primary care settings in England. An accurate list identifying the number of clinicians is not available; however, utilising NHS Digital staffing data (NHS Digital, 2024a, 2024b), it was estimated that a pool of approximately 48,000 clinicians were eligible to participate (Supporting Information S1: Table S1). Assuming a normal distribution, a 5% margin of error and a confidence interval of 95% (Taherdoost 2017), a calculated sample size of 381 responses was required (Raosoft, n.d.).

Participants were invited to undertake the survey via a hyperlink. Professional bodies, specialist interest groups, and NHS bodies (e.g., integrated care boards, spinal operational delivery networks) were contacted to request that the hyperlink be advertised to their members. In addition, professional contacts, and social media were utilised to disseminate the survey hyperlink (Supporting Information S1: Table S2). The survey was open between 21‐10‐2024 and 24‐12‐2024 and follow‐up contact was made twice during this period. The geographical response rate was monitored throughout the recruitment period to optimise the survey’s reach across all 42 integrated care boards (ICB) in England.

### 2.4 Survey

Microsoft Forms was used to host the survey tool (Microsoft 2024). The questions were developed considering the constructs described in the RE‐AIM framework for evaluating the success of implementation strategies (RE‐AIM Improving Public Health Relevance and Population Health Impact, n.d.). Pilot testing was completed in collaboration with clinicians from primary care. Informed consent was electronically collected following access to a participant information sheet. A combination of open and closed, fixed order, and adaptive questioning was used (Supporting Information S1: Figure S1). This assisted the participant to navigate the survey, while minimising the number and complexity of questions. Three categories of questions were employed: (1) eligibility questions, (2) survey questions, and (3) demographic questions. Depending on the route the participant took, a maximum of 11 survey questions were posed. All questions were mandatory, with the ability to return to questions enabled. The survey was anonymous, and to promote this, tracking of participants was not employed (e.g., no internet protocol addresses were collected). Completion of the survey was voluntary, with no incentive offered.

### 2.5 Analysis

Analysis was completed using Microsoft Excel (Office 365). As all questions were compulsory, with no data collected until the participants submitted their responses, no missing data was experienced. Descriptive analysis was employed to explore the aim. Frequency for numerical and Likert scale data was conveyed in absolute number (*n* =) and percentages (%).

## 3 Results

For clarity in this paper, national and regional CES guidelines and pathways will be referred to as ‘guidelines’, and locally agreed CES pathways will be referred to as ‘pathways’.

The survey was open for 64 days, with an average completion time of 12:53 (mm:ss). In total, 524 clinicians responded; however, nine were ineligible (Table 1), leaving 515 completed surveys. The majority of the participants identified as women (*n* = 262, 51%), were located across the seven NHS regions in England, with 11–16 years clinical experience, and most participants were First Contact Practitioners (FCP) (*n* = 251, 49%) (Table 2).

**TABLE 1:** Reasons for ineligibility to undertake survey.

	*n* = 9
Do not work in England	*n* = 4
Do not consent to take part	*n* = 2
Do not work in a primary care setting	*n* = 2
Do not assess or treat back pain in their job role	*n* = 1

**TABLE 2:** Demographic characteristics of respondents.

Characteristic (*n* = 515)	*n* = (%)
Gender	
Female	262 (51)
Male	240 (47)
Prefer not to say	13 (3)
NHS region in England	
South West	174 (34)
North West	79 (15)
North East and Yorkshire	73 (14)
Midlands	72 (14)
South East	57 (11)
London	38 (7)
East of England	22 (4)
Professional background	
First contact practitioner	251 (49)
General practitioner	108 (21)
Advanced practitioner	98 (19)
Musculoskeletal physiotherapist	49 (10)
Trainee advanced clinical practitioner	3 (1)
Physician's assistant	2 (0)
Allied health professional consultant	2 (0)
Urgent care practitioner	1 (0)
General practitioner trainee	1 (0)
Advanced practitioner and first contact practitioner professional background (*n* = 349)	
Physiotherapist	305 (87)
Paramedic	23 (7)
Nurse	17 (5)
Pharmacist	2 (1)
Osteopath	1 (0)
Other	1 (0)
Experience in years	
0–5	31 (6)
6–10	84 (16)
11–16	118 (23)
16–20	99 (19)
21–25	86 (17)
26–30	38 (7)
31–35	35 (7)
36–40	11 (2)
41–45	11 (2)
46–50	1 (0)
51+	1 (0)

### 3.1 Overall Awareness

Of the 515 participants, 452 (88%) were aware of any published CES guideline or pathway (Supporting Information S1: Table S3). The top three guidelines or pathways mentioned were: National Institute for Health and Care Excellence Low back pain and sciatica in over 16s: assessment and management (NICE guideline NG59) (*n* = 344, 67%), local cauda equina syndrome pathways (*n* = 315, 61%), and GIRFT: National Suspected Cauda Equina Syndrome Pathway (*n* = 297, 58%).

Among the top three professional groups by response number, those more likely to be aware of the GIRFT guidelines were FCPs (*n* = 168, 74%) and Advanced Practitioners (AP) (*n* = 61, 62%), whereas General Practitioners (GPs) (*n* = 10, 9%) were least likely (Table 3).

**TABLE 3:** Awareness of Getting It Right First Time (GIRFT): Spinal Surgery: National Suspected Cauda Equina Syndrome Pathway by Professional background.

	Number of respondents by professional background	Awareness of any CES guideline or pathway	Awareness of the GIRFT guideline
	*n* =	*n (*%)	*n (*%)
First contact practitioner	251	243 (97)	186 (74)
General practitioner	108	68 (63)	10 (9)
Advanced practitioner	98	86 (88)	61 (62)
Musculoskeletal physiotherapist	49	47 (96)	37 (76)
Allied health professional consultant	2	2 (100)	2 (100)
Trainee advanced clinical practitioner	3	3 (100)	1 (33)
Physician’s assistant	2	1 (50)	0 (0)
General practitioner trainee	1	1 (100)	0 (0)
Urgent care practitioner	1	1 (100)	0 (0)
Total	515	452	297

### 3.2 Local CES Pathways

Of the 452 participants who were aware of any published CES guidelines, 304 (67%) had access to a local CES pathway. Formal ratification, via the relevant local organisational governance department, occurred for 213 (70%) of these pathways.

The majority of local CES pathways were introduced prior to the launch of the GIRFT guidelines in February 2023, (*n* = 132, 43%), while 61 (20%) were introduced after this date, with 111 (37%) participants indicating they were ‘unsure’ of their pathway’s implementation date. Formally ratified local CES pathways followed a similar pattern, with 98 (46%) implemented prior to the launch of the GIRFT guidelines, 43 (20%) following its launch, and 72, (34%) ‘unsure’ of an implementation date.

### 3.3 Utilisation and Adherence

For clinicians who did not have access to a local CES pathway, 105 (50%) utilised national or regional CES guidelines to support their clinical decision‐making. The top three guidelines mentioned were: GIRFT: National Suspected Cauda Equina Syndrome Pathway (*n* = 40, 38%), NICE Low back pain and sciatica guideline NG59 (*n* = 36, 34%), and regional cauda equina syndrome guidelines (*n* = 12, 11%).

Combining the clinicians who had access to a local CES pathway (*n* = 304) and those that utilised a national or regional CES guidelines (*n* = 105), GPs (*n* = 22, 55%) were more likely to ‘agree or strongly agree’ that they would deviate from CES guidelines or pathways, compared to FCPs (*n* = 76, 32%) and APs (*n* = 24, 31%) (Supporting Information S1: Table S4).

### 3.4 Referral Routes for Suspected CES

Emergency referrals were most commonly directed to an Emergency Department or on‐call orthopaedics/neurosurgery team (Table 4).

**TABLE 4:** Top referral routes for patients suspected of having CES in Primary Care.

Emergency referrals				
	Access to a local CES pathway (*n* = 304)			
	Pathway implemented prior to GIRFT (*n* = 132)	Pathway implemented after GIRFT (*n* = 61)	Unsure of pathway implementation date (*n* = 111)	No access to a local pathway (*n* = 105)
	*n* (%)	*n* (%)	*n* (%)	*n* (%)
Emergency department	102 (77)	51 (84)	88 (79)	86 (82)
On‐call orthopaedic or neurosurgery team	27 (20)	7 (11)	12 (10)	16 (15)

Urgent referrals				
	Access to a local CES pathway (*n* = 304)			
	Pathway implemented prior to GIRFT (*n* = 132)	Pathway implemented after GIRFT (*n* = 61)	Unsure of pathway implementation date (*n* = 111)	No access to a local pathway (*n* = 105)
	*n* (%)	*n* (%)	*n* (%)	*n* (%)
Emergency department	51 (39)	6 (10)	38 (34)	40 (38)
On‐call orthopaedic or neurosurgery team	11 (8)	6 (10)	9 (8)	28 (27)
Musculoskeletal triage service	44 (33)	35 (57)	29 (26)	17 (16)

Urgent referral routes were influenced by the availability of a local CES pathway, and the date that the pathway was implemented. If a referrer had access to a local CES pathway, and that pathway was implemented after the launch of the GIRFT guidelines, urgent referrals were more likely to be referred to a Musculoskeletal (MSK) Triage Service (*n* = 35, 57%), as recommended in the GIRFT guidelines. However, if no local CES pathway was available, or the local CES pathway was implemented prior to the launch of the GIRFT guidelines, urgent referrals were typically directed to the Emergency Department (*n* = 40, 38%), while referral to a MSK Triage Service was less common (*n* = 17, 16%) (Table 4).

### 3.5 Mapping of Responses, Awareness, and Access to a CES Pathway

The 515 participants were distributed across all 42 Integrated Care Boards (ICB) in England (Supporting Information S1: Table S5). The 452 participants with awareness of any published CES guidelines or pathways were spread across 41 of the ICBs. The same 41 ICBs had at least one local CES pathway implemented in their catchment, with 38 ICBs having a pathway that was formally ratified via a local governance process (Supporting Information S1: Table S6).

### 3.6 Clinician’s Opinion of a Local CES Pathway

Most participants acknowledged the benefit of having access to a local CES pathway, with 276/304 (91%) ‘agreeing or strongly agreeing’ that it supported their clinical decision‐making. This was comparable to the 105 participants who did not have access to a local CES pathway but utilised a published national or regional CES guidelines, with 94/105 (90%) ‘agreeing or strongly agreeing’ that the guidelines supported their clinical decision‐making. Of the 105 participants, most (n = 90, 86%) also ‘agreed or strongly agreed’ that if their workplace implemented a local CES pathway it would support their clinical decision‐making (Table 5).

**TABLE 5:** Cauda equina syndrome guidelines or pathway adherence and support to clinical decision‐making.

Patient with access to a locally agreed CES pathway (*n* = 304)					
	Strongly agree	Agree	Neither agree or disagree	Disagree	Strongly disagree
	*n* (%)	*n* (%)	*n* (%)	*n* (%)	*n* (%)
I can think of a scenario where I would deviate from my workplace CES pathway	11 (4)	82 (27)	59 (19)	102 (34)	50 (16)
My workplace CES pathway supports my clinical decision‐making	131 (43)	145 (48)	13 (4)	7 (2)	8 (3)

Patient with no access to a locally agreed CES pathway (*n* = 105)					
	Strongly agree	Agree	Neither agree or disagree	Disagree	Strongly disagree
	*n* (%)	*n* (%)	*n* (%)	*n* (%)	*n* (%)
I can think of a scenario where I would deviate from a published CES clinical practice guidelines or pathway	4 (4)	40 (38)	28 (27)	23 (22)	10 (10)
Published CES clinical practice guidelines and pathways support my clinical decision‐making	32 (30)	62 (59)	7 (7)	3 (3)	1 (1)
If my workplace implemented a ratified CES pathway I believe this would support my clinical decision‐making	46 (44)	44 (42)	11 (10)	2 (2)	2 (2)

## 4 Discussion

### 4.1 Summary

The aim of this study was to explore the awareness and utilisation of the GIRFT guidelines in a primary care setting. Although most participants were aware of a published CES guideline, under two thirds were aware of the most recent iteration from GIRFT; this included less than 1‐in‐10 GPs. The translation of the GIRFT guideline into local CES pathways is also limited, with the majority of CES pathways being implemented prior to the GIRFT guidelines' launch date, therefore not reflecting its recommendations. In addition, one third of participants were unaware of when their local CES pathway was implemented, suggesting an unfamiliarity with the paradigm shift in patient management introduced by the GIRFT guidelines. However, for participants who did have access to a local pathway that was implemented after the GIRFT guidelines' launch date, their practice aligned more closely with recommendations in the GIRFT guidelines. This suggests that implementing a local CES pathway that reflects the GIRFT guidelines can promote access to current best‐practice care for patients suspected of having CES in a primary care setting.

This study shows that the GIRFT guidelines are not consistently followed in a primary care setting; these findings are comparable with previous research that reports similar inconsistency in secondary care (Fountain et al. 2019; Higginson et al. 2020). This can be explained by the limited awareness of the GIRFT guidelines described in this study. These findings align with previous research, which has shown that insufficient awareness can be a barrier to guideline implementation and utilisation (Fischer et al. 2016; Jabbour et al. 2018; Sorondo et al. 2021). The varied referral routes for urgent patients, described in this study, demonstrate how a deficient translation of the GIRFT guidelines into clinical practice can impact patient care; with a research‐to‐practice gap of up to 16 years (Glover et al. 2018) this issue may continue for many years to come.

This study demonstrated that of the participants who did have access to a local CES pathway, only 1‐in‐5 had access to a pathway that aligned with the recommendations in the GIRFT guidelines. Clinical pathways are an effective method to operationalise guidelines into clinical practice (Allen et al. 2009), they enable national guidelines to be fitted into a local context (e.g., resources, staffing), and they could also help increase clinician awareness of national recommendations. Numerous studies have shown the positive impact of local CES pathways on patient care (Buell et al. 2019; Fraig et al. 2023; Graham and Madhavan 2021), streamlining the process and expediting access to radiology and surgery for this time sensitive condition. An inclusive CES pathway can also facilitate the transfer of patients from a primary care setting into a secondary care CES referral pathway (Gill et al. 2024), enabling a smooth transition across the sometimes troublesome primary care/secondary care interface. These findings demonstrate an opportunity for improvement in the management of patients suspected of having CES in primary care.

### 4.2 Strengths and Limitations

This study represents the first research into the awareness and use of CES guidelines in the primary care setting in England, providing novel insights into how national guidelines translate into clinical practice in this setting. Its findings are based on responses from clinicians in all 42 ICBs in England, demonstrating its wide reach into primary care.

Recognition of the study's limitations is important when interpreting the results. Although the online platform enabled wider participant access, it also introduced the risk of a digital divide. The use of a survey methodology limited the depth of data collected and therefore limited the depth of interpretation feasible. For example, assumptions were made that any pathway implemented following the launch of the GIRFT guidelines would reflect their recommendations, but this was not confirmed. The survey format can only report what participants ‘say they would do’ rather than ‘what they do’ in clinical practice, therefore introducing a risk of social desirability bias (Bispo Júnior 2022). Finally, a representative sample of clinicians working in primary care was not achieved. Engagement from professional bodies was limited, which reduced access to the complete sample frame. The majority of clinicians working in primary care are GPs, but the majority of participants responding to this survey were physiotherapists. Although this is not representative of clinicians working in primary care, the findings still demonstrate that within the participants who did respond, fewer than 1‐in‐10 GPs are aware of the GIRFT guidelines. Although this is not generalisable to all GPs, it identifies an area that requires additional investigation.

### 4.3 Implications for Practice and Research

The findings of this study support the adoption, or revision, of a local CES pathway that aligns with the GIRFT guidelines (Getting It Right First Time (GIRFT) 2023). Clinical pathways have the ability to improve outcomes for spinal patients (Boyle et al. 2021; Murphy et al. 2022) in addition to expediting patient care for suspected CES (Buell et al. 2019; Fraig et al. 2023; Gill et al. 2024; Graham and Madhavan 2021). This study suggests that implementing local CES pathways could also increase primary care's adherence to national CES guidelines, but the limited awareness in some professions needs to be overcome for maximum impact.

Future research should explore the underlying factors that influence the variability in awareness and translation of the GIRFT guidelines into the primary care setting, specifically with GPs. In addition, it is paramount to understand the impact of this variability on patient experience of a referral from primary care for suspected CES. Finally, with only 1‐in‐10 responding GPs being aware of the GIRFT guidelines, an investigation into the impact of the GIRFT guidelines on reducing unwarranted referrals for emergency assessment and investigation is needed.

## 5 Conclusion

This is the first study to investigate the awareness and utilisation of the GIRFT guidelines in primary care clinicians in England. This study has identified a limited awareness of the GIRFT guidelines in primary care, particularly with GPs. It has also identified a possible solution to this problem with the introduction of locally agreed CES pathways. Up‐to‐date pathways were shown to facilitate the adherence to the GIRFT guidelines, specifically for urgent patients, and further understanding of how to increase the utilisation of the GIRFT guidelines in primary care is warranted.

## Author Contributions

**Jonathon Gill:** conceptualisation, methodology, investigation, data curation, formal analysis, writing – original draft, writing – review and editing, project administration, funding acquisition. **Sue Greenhalgh:** conceptualisation, methodology, supervision, formal analysis, writing – review and editing. **Jos M. Latour:** conceptualisation, methodology, supervision, formal analysis, writing – review and editing. **Gillian Yeowell:** conceptualisation, methodology, supervision, formal analysis, writing – review and editing.

## Conflicts of Interest

The authors declare no conflicts of interest.

## Supporting information

Supporting Information S1

Figure S1

## Data Availability

An a priori study protocol and open access data are available on the Open Science Framework at DOI https://doi.org/10.17605/OSF.IO/ECVUG.
