# Supplementary material for: Cauda Equina Syndrome: A Survey of Guideline Utilisation in Primary Care in England
Source: Musculoskeletal Care. 2025 Jun 5;23(2):e70129. doi: 10.1002/msc.70129 (PMC12140976; doi:10.1002/msc.70129)
Supplement: Supplementary file 1 — Supporting Information S1 [file MSC-23-e70129-s002.docx]

# Supporting Information - Tables

Table S1: Primary Care staffing

| General Practitioner | 37041 |
| --- | --- |
| Physician Associate | 2004 |
| Advanced Nurse Practitioner | 4461 |
| Advanced Paramedic Practitioner | 535 |
| Advanced Physiotherapy Practitioner | 129 |
| First Contact Practitioner | 1587 |
| Paramedic | 1953 |
| Physiotherapist | 110 |
| **Total** | **47820** |
| NHS Digital 2024a, NHS Digital 2024b | |

Table S2: List of professional bodies and specialist interest groups to contact participants

| **Professional bodies**   - Royal College of General Practice - Chartered Society of Physiotherapy - College of Paramedics - Royal College of Nursing   **Specialist Interest Groups**   - Advanced Practice Physiotherapy Network - Consultant Physiotherapist Professional Network - Musculoskeletal Associate of Chartered Physiotherapists - Society of Musculoskeletal Medicine - iCSP - MSK Partnership Group   **NHS Bodies**   - Integrated Care Boards - Local Medical Committees - GP Federations - NHS Spinal Operational Delivery Networks - Primary Care Commissioning Working Group   **Other Strategies**   - Personal NHS contacts (Research Team) - Social media e.g., X (formerly Twitter) |
| --- |

Table S3: Respondents awareness of clinical practice guidelines or pathways for the identification and early management of patients suspected of having cauda equina syndrome

| **Guideline** | **n=** |
| --- | --- |
| National Institute for Health and Care Excellence Low back pain and sciatica in over 16s: assessment and management (NICE guideline NG59) | 344 |
| Locally agreed cauda equina syndrome pathway | 315 |
| Getting It Right First Time (GIRFT): Spinal Surgery: National Suspected Cauda Equina Syndrome Pathway | 297 |
| NHS England: National Low Back and Radicular Pain Pathway | 244 |
| National Spinal Network: Early Recognition of Cauda Equina Syndrome: A Framework for Assessment and Referral for Primary care / MSK interface services | 155 |
| British Association of Spine Surgeons and Society of British Neurosurgeons: Standards of care for investigation and management of cauda equina syndrome | 142 |
| Regionally agreed cauda equina syndrome pathway | 132 |
| Royal College of General Practitioners: Clinical guidelines for the management of acute low back pain | 113 |
| WHO guideline for non-surgical management of chronic primary low back pain in adults in primary and community care settings | 59 |
| Royal College of Radiologists: MRI provision for cauda equina syndrome | 44 |
| The Royal College of Emergency Medicine: Position Statement Cauda Equina Syndrome | 37 |

Table S4: Deviation from local CES pathway, or national or regional CES guideline by profession

| I can think of a scenario where I would deviate from my workplace CES pathway | | | | | |
| --- | --- | --- | --- | --- | --- |
| **Professional Background**  **(n=304)** | **Strongly agree** | **Agree** | **Neither agree or disagree** | **Disagree** | **Strongly disagree** |
|  | **n= (%)** | **n= (%)** | **n= (%)** | **n= (%)** | **n= (%)** |
| First Contact Practitioner | 6 (3) | 49 (26) | 39 (20) | 63 (33) | 35 (18) |
| General Practitioner | 1 (9) | 5 (45) | 1 (9) | 4 (36) | 0 (0) |
| Advanced Practitioner | 2 (3) | 17 (29) | 14 (24) | 19 (32) | 7 (12) |
| Musculoskeletal Physiotherapist | 2 (5) | 11 (28) | 4 (10) | 15 (38) | 7 (18) |
| Allied Health Professional Consultant | 0 (0) | 0 (0) | 0 (0) | 1 (100) | 0 (0) |
| Trainee Advanced Clinical Practitioner | 0 (0) | 0 (0) | 1 (100) | 0 (0) | 0 (0) |
| Urgent Care Practitioner | 0 (0) | 0 (0) | 0 (0) | 0 (0) | 1 (100) |
| **Total** | **11 (4)** | **82 (27)** | **59 (19)** | **102 (34)** | **50 (16)** |
| I can think of a scenario where I would deviate from a published CES clinical practice guideline or pathway | | | | | |
| **Professional Background**  **(n=105)** | **Strongly agree** | **Agree** | **Neither agree or disagree** | **Disagree** | **Strongly disagree** |
|  | **n= (%)** | **n= (%)** | **n= (%)** | **n= (%)** | **n= (%)** |
| First Contact Practitioner | 2 (4) | 19 (42) | 10 (22) | 10 (22) | 4 (9) |
| General Practitioner | 2 (7) | 14 (48) | 7 (24) | 4 (14) | 2 (7) |
| Advanced Practitioner | 0 (0) | 5 (26) | 7 (37) | 6 (32) | 1 (5) |
| Musculoskeletal Physiotherapist | 0 (0) | 1 (13) | 2 (25) | 3 (38) | 2 (25) |
| Allied Health Professional Consultant | 0 (0) | 0 (0) | 1 (100) | 0 (0) | 0 (0) |
| Trainee Advanced Clinical Practitioner | 0 (0) | 0 (0) | 1 (100) | 0 (0) | 0 (0) |
| Physician’s Assistant | 0 (0) | 0 (0) | 0 (0) | 0 (0) | 1 (100) |
| General Practitioner trainee | 0 (0) | 1(100) | 0 (0) | 0 (0) | 0 (0) |
| **Total** | **4 (4)** | **40 (38)** | **28 (27)** | **23 (22)** | **10 (10)** |

Table S5: Number of responses be NHS Region and Integrated Care Boards (ICB)

| **East of England** | **22** |
| --- | --- |
| Bedfordshire, Luton and Milton Keynes ICB | 5 |
| Cambridgeshire and Peterborough ICB | 4 |
| Hertfordshire and West Essex ICB | 2 |
| Mid and South Essex ICB | 3 |
| Norfolk and Waveney ICB | 7 |
| Suffolk and North East Essex ICB | 1 |
| **London** | **38** |
| North Central London ICB | 7 |
| North East London ICB | 8 |
| North West London ICB | 9 |
| South East London ICB | 10 |
| South West London ICB | 4 |
| **Midlands** | **72** |
| Birmingham and Solihull ICB | 10 |
| Black Country ICB | 10 |
| Coventry and Warwickshire ICB | 9 |
| Derby and Derbyshire ICB | 11 |
| Herefordshire and Worcestershire ICB | 2 |
| Leicester, Leicestershire and Rutland ICB | 5 |
| Lincolnshire ICB | 12 |
| Northamptonshire ICB | 1 |
| Nottingham and Nottinghamshire ICB | 1 |
| Shropshire, Telford and Wrekin ICB | 8 |
| Staffordshire and Stoke-on-Trent ICB | 3 |
| **North East and Yorkshire** | **73** |
| Humber and North Yorkshire ICB | 17 |
| North East and North Cumbria ICB | 18 |
| South Yorkshire ICB | 5 |
| West Yorkshire ICB | 33 |
| **North West** | **79** |
| Cheshire and Merseyside ICB | 33 |
| Greater Manchester ICB | 34 |
| Lancashire and South Cumbria ICB | 12 |
| **South East** | **57** |
| Buckinghamshire, Oxfordshire and Berkshire West ICB | 5 |
| Frimley ICB | 5 |
| Hampshire and Isle of Wight ICB | 22 |
| Kent and Medway ICB | 2 |
| Surrey Heartlands ICB | 8 |
| Sussex ICB | 15 |
| **South West** | **174** |
| Bath and North East Somerset, Swindon and Wiltshire ICB | 11 |
| Bristol, North Somerset and South Gloucestershire ICB | 14 |
| Cornwall and The Isles of Scilly ICB | 37 |
| Devon ICB | 50 |
| Dorset ICB | 15 |
| Gloucestershire ICB | 8 |
| Somerset ICB | 39 |

Table S6: Mapping of awareness of CES guidelines or pathways, access to a local CES pathway, and ratification of local CES pathways

|  | **Awareness of CES guidelines or pathways** | **Access to a local CES pathway** | **Number of local CES pathways ratified** |
| --- | --- | --- | --- |
|  | n= | n= | n= |
| **East of England** |  |  |  |
| Bedfordshire, Luton and Milton Keynes ICB | 5 | 4 | 2 |
| Cambridgeshire and Peterborough ICB | 4 | 2 | 1 |
| Hertfordshire and West Essex ICB | 2 | 2 | 1 |
| Mid and South Essex ICB | 3 | 2 | 1 |
| Norfolk and Waveney ICB | 6 | 3 | 1 |
| Suffolk and North East Essex ICB | 1 | 1 | 0 |
| **London** |  |  |  |
| North Central London ICB | 6 | 4 | 3 |
| North East London ICB | 8 | 6 | 5 |
| North West London ICB | 9 | 9 | 5 |
| South East London ICB | 7 | 2 | 1 |
| South West London ICB | 4 | 3 | 3 |
| **Midlands** |  |  |  |
| Birmingham and Solihull ICB | 8 | 7 | 4 |
| Black Country ICB | 10 | 9 | 5 |
| Coventry and Warwickshire ICB | 9 | 4 | 2 |
| Derby and Derbyshire ICB | 9 | 6 | 5 |
| Herefordshire and Worcestershire ICB | 2 | 0 | 0 |
| Leicester, Leicestershire and Rutland ICB | 5 | 3 | 2 |
| Lincolnshire ICB | 10 | 5 | 0 |
| Northamptonshire ICB | 1 | 1 | 1 |
| Nottingham and Nottinghamshire ICB | 1 | 1 | 0 |
| Shropshire, Telford and Wrekin ICB | 8 | 3 | 1 |
| Staffordshire and Stoke-on-Trent ICB | 3 | 2 | 2 |
| **North East and Yorkshire** |  |  |  |
| Humber and North Yorkshire ICB | 15 | 10 | 5 |
| North East and North Cumbria ICB | 17 | 14 | 13 |
| South Yorkshire ICB | 5 | 5 | 5 |
| West Yorkshire ICB | 27 | 24 | 17 |
| **North West** |  |  |  |
| Cheshire and Merseyside ICB | 19 | 11 | 8 |
| Greater Manchester ICB | 33 | 24 | 17 |
| Lancashire and South Cumbria ICB | 11 | 4 | 4 |
| **South East** |  |  |  |
| Buckinghamshire, Oxfordshire and Berkshire West ICB | 4 | 3 | 2 |
| Frimley ICB | 5 | 2 | 1 |
| Hampshire and Isle of Wight ICB | 17 | 12 | 6 |
| Kent and Medway ICB | 2 | 2 | 2 |
| Surrey Heartlands ICB | 8 | 8 | 4 |
| Sussex ICB | 15 | 11 | 7 |
| **South West** |  |  |  |
| Bath and North East Somerset, Swindon and Wiltshire ICB | 11 | 9 | 6 |
| Bristol, North Somerset and South Gloucestershire ICB | 14 | 13 | 13 |
| Cornwall and The Isles of Scilly ICB | 33 | 16 | 10 |
| Devon ICB | 38 | 18 | 12 |
| Dorset ICB | 14 | 8 | 7 |
| Gloucestershire ICB | 7 | 3 | 1 |
| Somerset ICB | 36 | 28 | 28 |

Figure S1: Cauda equina syndrome guidelines in primary care survey question template

References:

NHS Digital. (2024a). *Primary care workforce*. Available from: <https://view.officeapps.live.com/op/view.aspx?src=https%3A%2F%2Ffiles.digital.nhs.uk%2F16%2F21B4CC%2FPrimary%2520Care%2520Workforce%252C%2520England%2520-%2520FTE%2520Nurses%252C%2520DPC%2520and%2520Admin%252C%2520June%25202024.xlsx&wdOrigin=BROWSELINK>. (Accessed: 27 August 2024).

NHS Digital. (2024b). General practice workforce. Available from: <https://view.officeapps.live.com/op/view.aspx?src=https%3A%2F%2Ffiles.digital.nhs.uk%2FD2%2FB428AB%2FGPW%2520Bulletin%2520Tables%2520-%2520June%25202024.xlsx&wdOrigin=BROWSELINK> (Accessed: 27 August 2024).
