# Supplementary material for: Cauda Equina Syndrome: A Survey of Guideline Utilisation in Primary Care in England
Source: Musculoskeletal Care. 2025 Jun 5;23(2):e70129. doi: 10.1002/msc.70129 (PMC12140976; doi:10.1002/msc.70129)
Supplement: Supplementary file 2 — Figure S1 [file MSC-23-e70129-s001.pdf]

Cauda Equina Syndrome Guidelines in Primary Care

IRAS: 347437; Version: v1; Date: 23 10 2024

Invitation to research
We would like to invite you to take part in our research on **Cauda Equina Syndrome (CES) guidelines and Primary Care**. My name is Jonathon Gill, and I am completing this research as part of my NIHR Pre-doctoral Clinical and Practitioner Academic Fellowship (PCAF), supervised by Professor Gillian Yeowell, Professor Jos Latour and Dr Sue Greenhalgh.

This survey aims to explore the uptake and utilisation of clinical practice guidelines for suspected CES in the NHS Primary Care setting in England.

Why have I been invited?
We are asking **NHS clinicians** who work in the **Primary Care setting** (either full or part-time) and **see patients with back pain**, as part of their Primary Care role, to undertake this survey. Our goal is to gather information from across all 42 Integrated Care Boards (ICBs) in England, and your help with this will be greatly appreciated.

Who has reviewed this research project?
This research has been reviewed by the Research Department at Somerset NHS Foundation Trust. It has gained NHS Health and Care Research Wales (HCRW) ethical and Health Research Authority approval - Ref: 24/HRA/3406.

The survey takes most people **less than 10 minutes** to complete, with the main section asking no more than 11 simple questions. It is completed via MS Forms, which meets GDPR compliance requirements. The anonymous data will be aggregated and securely stored in encrypted servers. The data analysed for publication will be stored on an open repository (OSF); however, this will be anonymous with limited demographic data uploaded to ensure participants are not identifiable. As the survey is anonymous, once submitted, participants responses cannot be identified or withdrawn.

If you would like further information please review the full Participant Information Sheet, which will be opened in a new window, by clicking on this link: **https://forms.office.com/e/zLkNhg6UGF**

If you are happy to proceed without further details, please complete the 4 eligibility questions below. Thank you for taking the time to complete this survey, your help is greatly appreciated.

* Required

1

Please confirm you have read the introductory information and wish to consent to take part in the survey *

◯ Yes

◯ No

2

Does any part of your job role occur in the Primary Care setting *

◯ Yes

◯ No

3

Does this part of your job role involve seeing people with back pain *

- Yes
- No

4

Is which Country is your Primary Care role based *

- England
- Scotland
- Wales
- Northern Ireland
- Ireland
- Other

Section

5

Are you aware of any national, regional or local clinical practice guidelines or pathways for the identification and early management of patients suspected of having cauda equina syndrome
*

- ○ Yes
- ○ No

6

From the list below, please identify all the clinical practice guidelines or pathways you **are aware** of for the identification and early management of patients suspected of having cauda equina syndrome (Please tick any you **are aware** of)
*

- ☐ British Association of Spine Surgeons and Society of British Neurosurgeons: Standards of care for investigation and management of cauda equina syndrome
- ☐ Royal College of General Practitioners: Clinical guidelines for the management of acute low back pain
- ☐ NHS England: National Low Back and Radicular Pain Pathway
- ☐ The Royal College of Emergency Medicine: Position Statement Cauda Equina Syndrome
- ☐ Getting It Right First Time: Spinal Surgery: National Suspected Cauda Equina Syndrome Pathway
- ☐ Royal College of Radiologists: MRI provision for cauda equina syndrome
- ☐ National Spinal Network: Early Recognition of Cauda Equina Syndrome: A Framework for Assessment and Referral for Primary care / MSK interface services
- ☐ Regionally agreed cauda equina syndrome pathway
- ☐ WHO guideline for non-surgical management of chronic primary low back pain in adults in primary and community care settings
- ☐ National Institute for Health and Care Excellence Low back pain and sciatica in over 16s: assessment and management (NICE guideline NG59)
- ☐ Locally agreed cauda equina syndrome pathway

n.b. 'Clinical practice guideline or pathway for managing patients suspected of having cauda equina syndrome' will now be referred to as: '**CES guideline**' or **'CES pathway**'

7

Has your workplace implemented a CES pathway?

*

- ◯ Yes
- ◯ No
- ◯ Currently being developed

Section

8

Ratified means, a document that has been officially approved by the relevant organisational governance departments in your workplace (e.g., Governance Support Team).

Is your workplace CES pathway ratified?

*

- ◯ Yes
- ◯ No

9

Who implemented your workplace CES Pathway? *

- ☐ The GP practice
- ☐ The Primary Care Network
- ☐ The MSK Triage Service
- ☐ The Secondary Care NHS Trust
- ☐ Unsure
- ☐ Other

10

To your knowledge, when did your workplace implement their CES pathway *

- ◯ Before March 2023
- ◯ March 2023 or after
- ◯ Unsure

11

Typically, CES pathways suggest referral route/s for patients needing further clinical assessment and/or investigation. For referrals from Primary Care, which referral route/s does your workplace CES pathway include?

(Tick all that apply) *

- ☐ Urgent (as soon as possbile)
- ☐ Routine
- ☐ Emergency (same day)
- ☐ No referral routes suggested

Section

Emergency is defined as: ongoing CES symptoms or signs which started, or have progressed, within the last two weeks.
Urgent is defined as: static CES symptoms for two weeks or more, or isolated bilateral lower limb symptoms or signs.

12

From Primary Care, which department or team do **emergency** patients suspected of having CES get referred to

(Tick the one that is most relevant) *

- First Contact Practitioner (FCP)
- Secondary Care Spinal Clinic
- MSK Triage Service
- On-call Orthopaedics or Neurosurgery team
- MSK Physiotherapy
- Emergency Department
- Other

13

From Primary Care, which department or team do **urgent** patients suspected of having CES get referred to

(Tick the one that is most relevant) *

- First Contact Practitioner (FCP)
- Secondary Care Spinal Clinic
- MSK Triage Service
- On-call Orthopaedics or Neurosurgery team
- MSK Physiotherapy
- Emergency Department
- Other

14

Which of these symptoms and signs feature in your workplace CES pathway

(Please tick all that apply) *

- [ ] Lower limb sensory changes
- [ ] Spinal range of movement
- [ ] Plantar response (Babinski response)
- [ ] Sexual dysfunction
- [ ] Bowel dysfunction
- [ ] Clonus
- [ ] Static symptoms
- [ ] Lower limb weakness
- [ ] Objective saddle anaesthesia
- [ ] Unilateral lower limb pain
- [ ] Muscle tone
- [ ] Subjective saddle anaesthesia
- [ ] Bilateral lower limb pain
- [ ] Hoffman's response
- [ ] Back pain
- [ ] Digital rectal examination
- [ ] Duration of symptoms
- [ ] Functional tests
- [ ] Altered reflexes
- [ ] Bladder dysfunction
- [ ] Gait disturbance

15

To what extent do you agree or disagree with the statement: *

	Strongly disagree	Disagree	Neither agree or disagree	Agree	Strongly agree
My workplace CES pathway supports my clinical decision making	○	○	○	○	○
I can think of a scenario where I would deviate from my workplace CES pathway	○	○	○	○	○

Section

16

For patients you suspect of having CES, do you use a CES guideline or pathway to support your clinical decision making? *

- ◯ Yes
- ◯ No

17

For patients you suspect of having CES, please identify from the list below, which clinical practice guideline or pathway most influences your clinical decision making
(Please tick one)
*

- ◯ British Association of Spine Surgeons and Society of British Neurosurgeons: Standards of care for investigation and management of cauda equina syndrome
- ◯ Royal College of General Practitioners: Clinical guidelines for the management of acute low back pain
- ◯ NHS England: National Low Back and Radicular Pain Pathway
- ◯ The Royal College of Emergency Medicine: Position Statement Cauda Equina Syndrome
- ◯ Getting It Right First Time: Spinal Surgery: National Suspected Cauda Equina Syndrome Pathway
- ◯ Royal College of Radiologists: MRI provision for cauda equina syndrome
- ◯ National Spinal Network: Early Recognition of Cauda Equina Syndrome: A Framework for Assessment and Referral for Primary care / MSK interface services
- ◯ Regionally agreed cauda equina syndrome pathway
- ◯ WHO guideline for non-surgical management of chronic primary low back pain in adults in primary and community care settings
- ◯ National Institute for Health and Care Excellence Low back pain and sciatica in over 16s: assessment and management (NICE guideline NG59)

18

Typically, CES guidelines suggest a referral route/s for patients needing further clinical assessment and/or investigation. For referrals from Primary Care, which referral route/s do you use with patients you suspect of having CES?

(Tick all that apply)
*

- ☐ Urgent (as soon and possible)
- ☐ Routine
- ☐ Emergency (same day)

Section

Emergency defined as: ongoing CES symptoms or signs which started, or have progressed, within the last two weeks.

Urgent is defined as: static CES symptoms for two weeks or more, or isolated bilateral lower limb symptoms or signs.

19

From Primary Care, which department or team do you refer **emergency** patients you suspect of having CES

(Tick the one that is most relevant) *

- First Contact Practitioner (FCP)
- Secondary Care Spinal Clinic
- MSK Triage Service
- On-call Orthopaedics or Neurosurgery team
- MSK Physiotherapy
- Emergency Department
- Other

20

From Primary Care, which department or team do you refer **urgent** patients you suspect of having CES

(Tick the one that is most relevant) *

- First Contact Practitioner (FCP)
- Secondary Care Spinal Clinic
- MSK Triage Service
- On-call Orthopaedics or Neurosurgery team
- MSK Physiotherapy
- Emergency Department
- Other

21

Which of these symptoms and signs do you most consider when assessing a patient you suspect of having CES

(Please tick all that apply) *

- [ ] Lower limb sensory changes
- [ ] Spinal range of movement
- [ ] Plantar response (Babinski response)
- [ ] Sexual dysfunction
- [ ] Bowel dysfunction
- [ ] Clonus
- [ ] Static symptoms
- [ ] Lower limb weakness
- [ ] Objective saddle anaesthesia
- [ ] Unilateral lower limb pain
- [ ] Muscle tone
- [ ] Subjective saddle anaesthesia
- [ ] Bilateral lower limb pain
- [ ] Hoffman’s response
- [ ] Back pain
- [ ] Digital rectal examination
- [ ] Duration of symptoms
- [ ] Functional tests
- [ ] Altered reflexes
- [ ] Bladder dysfunction
- [ ] Gait disturbance

22

To what extent do you agree or disagree with the statement: *

	Strongly disagree	Disagree	Neither agree or disagree	Agree	Strongly agree
Published CES clinical practice guidelines and pathways support my clinical decision making	○	○	○	○	○
I can think of a scenario where I would deviate from a published CES clinical practice guideline or pathway	○	○	○	○	○
If my workplace implemented a ratified CES pathway I believe this would support my clinical decision making	○	○	○	○	○

Demographic information

Could you please answer a few anonymous questions about yourself?

23

Which best describes your professional background *

- General Practitioner (GP)
- Doctor (non-GP)
- Physician's Assistant
- MSK Physiotherapist
- First Contact Practitioner (FCP)
- Advanced Practitioner
- Other

24

What was your primary healthcare role (FCP) *

- Physiotherapist
- Paramedic
- Nurse
- Pharmacologist
- Podiatrist
- Other

25

What was your primary healthcare role (AP) *

- ◯ Physiotherapist
- ◯ Paramedic
- ◯ Nurse
- ◯ Pharmacologist
- ◯ Podiatrist
- ◯ Other

26

Number of years since professional qualification as a medical/healthcare professional was obtained (please round up to the next whole year) *

- ◯ 0-5 years
- ◯ 6-10 years
- ◯ 11-15 years
- ◯ 16-20 years
- ◯ 21-25 years
- ◯ 26-30 years
- ◯ 31-35 years
- ◯ 36-40 years
- ◯ 41-45 years
- ◯ 46-50 years
- ◯ 51+ years

27

How do you currently describe your gender? *

- ◯ A man
- ◯ A woman
- ◯ Non-binary
- ◯ Prefer to self-describe
- ◯ Prefer not to say

Demographic information

Could you please answer a few anonymous questions about yourself?

28

Which best describes your professional background *

- General Practitioner (GP)
- Doctor (non-GP)
- Physician's Assistant
- MSK Physiotherapist
- First Contact Practitioner (FCP)
- Advanced Practitioner
- Other

29

What was your primary healthcare role (FCP) *

- Physiotherapist
- Paramedic
- Nurse
- Pharmacologist
- Podiatrist
- Other

30

What was your primary healthcare role (AP) *

- Physiotherapist
- Paramedic
- Nurse
- Pharmacologist
- Podiatrist
- Other

31

Number of years since professional qualification as a medical/healthcare professional was obtained (please round up to the next whole year) *

- 0-5 years
- 6-10 years
- 11-15 years
- 16-20 years
- 21-25 years
- 26-30 years
- 31-35 years
- 36-40 years
- 41-45 years
- 46-50 years
- 51+ years

32

How do you currently describe your gender? *

- A man
- A woman
- Non-binary
- Prefer to self-describe
- Prefer not to say

NHS Region

33

Which is your NHS Region in England *

- ◯ East of England
- ◯ London
- ◯ Midlands
- ◯ North East and Yorkshire
- ◯ North West
- ◯ South East
- ◯ South West

East of England Primary Care Networks

34

Which is your Integrated Care Board (ICB) in East of England *

- NHS Bedfordshire, Luton and Milton Keynes ICB
- NHS Cambridgeshire and Peterborough ICB
- NHS Hertfordshire and West Essex ICB
- NHS Mid and South Essex ICB
- NHS Norfolk and Waveney ICB
- NHS Suffolk and North East Essex ICB

35

Which is your Primary Care Network (PCN) in NHS Bedfordshire, Luton and Milton Keynes ICB *

- ASCENT PCN
- CARITAS MEDICAL PCN
- CHILTERN HILLS PCN
- CROWN PCN
- EAST BEDFORD PCN
- EAST MK PCN
- EQUALITY PCN
- GREEN VALE HEALTH PCN
- HATTERS HEALTH PCN
- HILLTON PCN
- IVEL VALLEY SOUTH PCN
- LEA VALE PCN
- LEIGHTON LINSLADE HEALTH CONNECTIONS PCN
- MEDICS PCN
- NEXUS MK PCN
- NORTH BEDFORD PCN
- OASIS PCN
- ONEMK PCN
- PHOENIX SUNRISERS PCN
- SANDHILLS PCN
- SOUTH WEST PCN
- THE BRIDGE MK PCN
- TITAN PCN
- UNITY (BEDFORD) PCN
- WATLING STREET NETWORK PCN
- Other

36

Which is your Primary Care Network (PCN) in NHS Cambridgeshire and Peterborough ICB *

- A1 NETWORK PCN
- BMC PASTON PCN
- BRETTON PARK & HAMPTON PCN
- CAM MEDICAL PCN
- CAMBRIDGE CITY 4 PCN
- CAMBRIDGE CITY PCN
- CAMBRIDGE NORTH VILLAGES PCN
- CANTAB MEDICAL PRACTICES PCN
- CENTRAL THISTLEMOOR & THORPE PCN
- ELY NORTH PCN
- ELY SOUTH PCN
- FENLAND PCN
- GRANTA PCN
- HUNTINGDON PCN
- MERIDIAN PCN
- PETERBOROUGH & EAST PCN
- PETERBOROUGH PARTNERSHIPS PCN
- SOUTH FENLAND PCN
- SOUTH PETERBOROUGH PCN
- ST IVES PCN
- ST NEOTS PCN
- WISBECH PCN
- Other

37

Which is your Primary Care Network (PCN) in NHS Hertfordshire and West Essex ICB *

- ABBEY HEALTH PCN
- ALBAN HEALTHCARE PCN
- ALLIANCE PCN
- ALPHA PCN
- ATTENBOROUGH PCN
- BRIDGEWATER PCN
- BROXBOURNE ALLIANCE PCN
- CENTRAL WATFORD PCN
- DACORUM BETA PCN
- DELTA PCN
- EPPING FOREST NORTH PCN
- GRAND UNION PCN
- HALO PCN
- HARLOW NORTH PCN
- HARLOW SOUTH PCN
- HARPENDEN HEALTH PCN
- HATFIELD PCN
- HERTFORD AND RURALS PCN
- HERTS FIVE PCN
- HITCHIN AND WHITWELL PCN
- HODDESDON & BROXBOURNE PCN
- ICKNIELD PCN
- LEA VALLEY HEALTH PCN
- LOUGHTON BUCKHURST HILL & CHIGWELL PCN
- MVPS PCN
- NORTH UTTLESFORD PCN
- NORTH WATFORD PCN
- POTTERS BAR PCN
- RICKMANSWORTH & CHORLEYWOOD PCN

- ◯ SOUTH UTTLESFORD PCN
- ◯ STEVENAGE NORTH PCN
- ◯ STEVENAGE SOUTH PCN
- ◯ STORT VALLEY & VILLAGES PCN
- ◯ WARE AND RURALS PCN
- ◯ WELWYN GARDEN CITY A PCN
- ◯ Other

38

Which is your Primary Care Network (PCN) in NHS Mid and South Essex ICB *

- AEGROS HEALTH PCN
- ASOP PCN
- BENFLEET PCN
- BILLERICAY PCN
- BRAINTREE PCN
- BRENTWOOD PCN
- CANVEY PCN
- CENTRAL BASILDON PCN
- CHELMER PCN
- CHELMSFORD CITY HEALTH PCN
- CHELMSFORD WEST PCN
- COLNE VALLEY PCN
- DENGIE & SOUTH WOODHAM FERRERS PCN
- EAST BASILDON PCN
- GRAYS PCN
- MALDON & WITHAM PCN
- RAYLEIGH AND DISTRICT PCN
- ROCHFORD PCN
- SOUTHEND EAST PCN
- SOUTHEND VICTORIA PCN
- SOUTHEND WEST CENTRAL PCN
- SS9 PCN
- STANFORD-LE-HOPE PCN
- TILBURY AND CHADWELL PCN
- WEST BASILDON PCN
- WICKFORD PCN
- Other

39

Which is your Primary Care Network (PCN) in NHS Norfolk and Waveney ICB *

- BRECKLAND SURGERIES PCN
- CENTRAL NORWICH PCN
- FENS & BRECKS PCN
- GORLESTON PCN
- GREAT YARMOUTH & NORTHERN VILLAGES PCN
- KETTS OAK PCN
- KINGS LYNN PCN
- LOWESTOFT PCN
- MID NORFOLK PCN
- NORTH NORFOLK 1 PCN
- NORTH NORFOLK 2 PCN
- NORTH NORFOLK 3 PCN
- NORTH NORFOLK 4 PCN
- NORWICH EAST PCN
- NORWICH NORTH PCN
- NORWICH PCN
- SOUTH NORFOLK HIP PCN
- SOUTH WAVENEY PCN
- SWAFFHAM & DOWNHAM MARKET PCN
- WEST NORFOLK COASTAL PCN
- WEST NORWICH PCN
- Other

40

Which is your Primary Care Network (PCN) in NHS Suffolk and North East Essex ICB *

- BARRACK LANE & IVRY STREET PCN
- BLACKBOURNE PCN
- BURY ST EDMUNDS PCN
- CLACTON PCN
- COLCHESTER MEDICAL GROUP PCN
- COLTE PARTNERSHIP (ARA) PCN
- COLTE PARTNERSHIP (TMR) PCN
- COLTE PARTNERSHIP (WCW) PCN
- CREFFIELD MEDICAL GROUP PCN
- EAST HILL, ABBEY FIELDS & TOLLGATE PCN
- EAST IPSWICH PCN
- EAST SUFFOLK PCN
- FOREST HEATH PCN
- HAVERHILL PCN
- NORTH COLCHESTER PCN
- NORTH EAST COASTAL PCN
- NORTH EAST IPSWICH PCN
- ORWELL PCN
- RANWORTH PCN
- SOUTH RURAL PCN
- SUDBURY PCN
- TENDRING PCN
- THE DEBEN HEALTH GROUP PCN
- WGGL PCN
- WOLSEY PCN
- Other

London Primary Care Networks

41

Which is your Integrated Care Board (ICB) in London *

- NHS North Central London ICB
- NHS North East London ICB
- NHS North West London ICB
- NHS South East London ICB
- NHS South West London ICB

42

Which is your Primary Care Network (PCN) in NHS North Central London ICB *

- BARNET 1D PCN
- BARNET 1W PCN
- BARNET 2 PCN
- BARNET 3 PCN
- BARNET 4 PCN
- BARNET 5 PCN
- BARNET 6 PCN
- CENTRAL 1 ISLINGTON PCN
- CENTRAL 2 ISLINGTON PCN
- CENTRAL CAMDEN PCN
- CENTRAL HAMPSTEAD PCN
- EDMONTON PCN
- ENFIELD CARE NETWORK PCN
- ENFIELD SOUTH WEST PCN
- ENFIELD UNITY PCN
- HARINGEY - EAST CENTRAL PCN
- HARINGEY - N15/SOUTH EAST PCN
- HARINGEY - NORTH CENTRAL PCN
- HARINGEY - NORTH EAST PCN
- HARINGEY - NORTH WEST PCN
- HARINGEY - SOUTH WEST PCN
- HARINGEY - WELBOURNE PCN
- KENTISH TOWN CENTRAL PCN
- KENTISH TOWN SOUTH PCN
- NORTH 1 ISLINGTON PCN
- NORTH 2 ISLINGTON PCN
- NORTH CAMDEN PCN
- SOUTH CAMDEN PCN
- SOUTH ISLINGTON PCN

- ◯ WEST AND CENTRAL PCN
- ◯ WEST CAMDEN PCN
- ◯ WEST ENFIELD COLLABORATIVE PCN
- ◯ Other

43

Which is your Primary Care Network (PCN) in NHS North East London ICB *

- BARKING & DAGENHAM EAST ONE PCN
- BARKING & DAGENHAM EAST PCN
- BARKING & DAGENHAM NEW WEST PCN
- BARKING & DAGENHAM NORTH PCN
- BARKING & DAGENHAM NORTH WEST PCN
- BARKING & DAGENHAM WEST PCN
- BROMLEY BY BOW AND STEPNEY HEALTH CIC
- CLISSOLD PARK PCN
- CRANBROOK PCN
- DOCKLANDS PCN
- E4 NETWORK PCN
- FAIRLOP PCN
- HACKNEY DOWNS PCN
- HACKNEY MARSHES PCN
- HAVERING CREST PCN
- HAVERING MARSHALL PCN
- HAVERING NORTH PCN
- HAVERING SOUTH PCN
- LONDON FIELDS PCN
- LOXFORD PCN
- NEW CROSS ALLIANCE PCN
- NEWHAM CENTRAL 1 PCN
- NEWHAM CENTRAL PCN
- NEWHAM NORTH EAST 1 PCN
- NEWHAM NORTH EAST 2 PCN
- NEWHAM NORTH WEST 2 PCN
- NORTH NEWHAM PCN
- SEVEN KINGS PCN
- SHOREDITCH PARK PCN

- SOUTH ONE NEWHAM PCN
- SPRINGFIELD PARK PCN
- STRATFORD PCN
- TOWER HAMLETS NETWORK 1 PCN
- TOWER HAMLETS NETWORK 2 PCN
- TOWER HAMLETS NETWORK 5 PCN
- TOWER HAMLETS NETWORK 7 PCN
- TOWER HAMLETS NETWORK 8 PCN
- TOWER NETWORK PCN
- WALTHAM FOREST 8 PCN
- WALTHAM FOREST INTEGRATED HEALTH PCN
- WALTHAM FOREST LEYTON COLLABORATIVE PCN
- WALTHAM FOREST SOUTH LEYTONSTONE PCN
- WALTHAM FOREST WALTHAMSTOW CENTRAL PCN
- WALTHAM FOREST WALTHAMSTOW WEST PCN
- WANSTEAD AND WOODFORD PCN
- WELL STREET COMMON PCN
- WOODBERRY WETLANDS PCN
- Other

44

Which is your Primary Care Network (PCN) in NHS North West London ICB *

- ACTON PCN
- BABYLON GP AT HAND PCN
- BRENT CENTRAL KWH PCN
- BRENT NORTH KWH PCN
- BRENT SOUTH KWH PCN
- BRENT WEST KWH PCN
- BRENTWORTH PCN
- BROMPTON HEALTH PCN
- CELADINE HEALTH & METROCARE PCN
- CHISWICK PCN
- COLNE UNION PCN
- FELTHAM AND BEDFONT PCN
- GREAT WEST ROAD PCN
- GREENWELL PCN
- HAMMERSMITH & FULHAM CENTRAL PCN
- HAMMERSMITH & FULHAM PARTNERSHIP PCN
- HARNESS NORTH PCN
- HARNESS SOUTH PCN
- HARROW COLLABORATIVE PCN
- HARROW EAST PCN
- HEALTH ALLIANCE PCN
- HEALTHSENSE PCN
- HH COLLABORATIVE PCN
- HOUNSLOW HEALTH PCN
- INCLUSIVE HEALTH PCN
- K AND C SOUTH PCN
- KILBURN PARTNERSHIP PCN
- LONG LANE FIRST CARE GROUP PCN
- NEOHEALTH PCN

- ◯ NGP PCN
- ◯ NORTH CONNECT PCN
- ◯ NORTH HAMMERSMITH & FULHAM PCN
- ◯ NORTH SOUTHALL PCN
- ◯ NORTHOLT PCN
- ◯ REGENT HEALTH PCN
- ◯ SOUTH CENTRAL EALING PCN
- ◯ SOUTH FULHAM PCN
- ◯ SOUTH SOUTHALL PCN
- ◯ SOUTH WESTMINSTER PCN
- ◯ SPHERE PCN
- ◯ ST JOHN'S WOOD & MAIDA VALE PCN
- ◯ SYNERGY PCN
- ◯ THE EALING NETWORK PCN
- ◯ WEST END & MARYLEBONE PCN
- ◯ WEST-HILL HEALTH PCN
- ◯ Other

45

Which is your Primary Care Network (PCN) in NHS South East London ICB *

- APL BEXLEY PCN
- APLOS HEALTH PCN
- AT MEDICS STREATHAM PCN
- BECKENHAM PCN
- BLACKHEATH AND CHARLTON PCN
- BRIXTON AND CLAPHAM PARK PCN
- BROMLEY CONNECT PCN
- CLAPHAM PCN
- CLOCKTOWER PCN
- CROXTED PCN
- ELTHAM PCN
- FIVE ELMS PCN
- FIVEWAYS PCN
- FROGNAL PCN
- GREENWICH WEST PCN
- HAYES WICK PCN
- HERITAGE PCN
- HILLS, BROOKS & DALES GROUP PCN
- LEWISHAM ALLIANCE PCN
- LEWISHAM CARE PARTNERSHIP PCN
- MODALITY LEWISHAM PCN
- MOTTINGHAM, DOWNHAM & CHISLEHURST PCN
- NORTH BEXLEY PCN
- NORTH LAMBETH PCN
- NORTH LEWISHAM PCN
- NORTH SOUTHWARK PCN
- ORPINGTON PCN
- PENGE PCN
- RIVERVIEW HEALTH PCN

- SEVENFIELDS PCN
- SOUTH SOUTHWARK PCN
- STOCKWELLBEING PCN
- STREATHAM PCN
- THE CRAYS COLLABORATIVE PCN
- UNITY (GREENWICH) PCN
- VALENTINE HEALTH PCN
- Other

46

Which is your Primary Care Network (PCN) in NHS South West London ICB *

- BALHAM, TOOTING & FURZEDOWN PCN
- BATTERSEA PCN
- BROCKLEBANK PCN
- CANBURY CHURCHILL ORCHARD BERRYLANDS PCN
- CARSHALTON PCN
- CENTRAL SUTTON PCN
- CHEAM AND SOUTH SUTTON PCN
- CHESSINGTON AND SURBITON PCN
- CROYDON GP SUPER NETWORK PCN
- CROYDON LINK PCN
- EAST MERTON PCN
- EAST TWICKENHAM PCN
- GPNET5 PCN
- GRAFTON MEDICAL PARTNERS PCN
- HAMPTON PCN
- KESTON MOORINGS & PARKSIDE PCN
- KINGSTON PCN
- MAYDAY SOUTH PCN
- MORDEN PCN
- NEW MALDEN & WORCESTER PARK PCN
- NIGHTINGALE PCN
- NORTH MERTON PCN
- NORTH WEST MERTON PCN
- ONE THORNTON HEATH PCN
- PRIMARY CARE NORTH CROYDON PCN
- PRIME WANDSWORTH PCN
- RICHMOND PCN
- SELSDON ADDINGTON & SHIRLEY PCN
- SELSDON PURLEY & COULSDON HEALTH PCN

- SHEEN & BARNES PCN
- SOUTH WEST MERTON PCN
- SURBITON HEALTH CENTRE PCN
- TEDDINGTON PCN
- WALLINGTON PCN
- WANDLE PCN
- WANDSWORTH PCN
- WEST MERTON PCN
- WEST TWICKENHAM PCN
- WEST WANDSWORTH PCN
- Other

Midlands Primary Care Networks

47

Which is your Integrated Care Board (ICB) in Midlands *

- NHS Birmingham and Solihull ICB
- NHS Black Country ICB
- NHS Coventry and Warwickshire ICB
- NHS Derby and Derbyshire ICB
- NHS Herefordshire and Worcestershire ICB
- NHS Leicester, Leicestershire and Rutland ICB
- NHS Lincolnshire ICB
- NHS Northamptonshire ICB
- NHS Nottingham and Nottinghamshire ICB
- NHS Shropshire, Telford and Wrekin ICB
- NHS Staffordshire and Stoke-on-Trent ICB

48

Which is your Primary Care Network (PCN) in NHS Birmingham and Solihull ICB *

- ALLIANCE OF SUTTON PRACTICES PCN
- BALSALL HEATH, SPARKHILL & MOSELEY PCN
- BIRMINGHAM EAST CENTRAL PCN
- BORDESLEY EAST PCN
- BOURNVILLE AND NORTHFIELD PCN
- COMMUNITY CARE HALL GREEN PCN
- EDGBASTON PCN
- GOSK PCN
- GPS HEALTHCARE PCN
- HARBORNE PCN
- KINGSTANDING, ERDINGTON & NECHELLS PCN
- MMP CENTRAL AND NORTH PCN
- MOSELEY, BILLESLEY & YARDLEY WOOD PCN
- NECHELLS, SALTLEY & ALUM ROCK PCN
- NORTH BIRMINGHAM PCN
- NORTH SOLIHULL PCN
- PEOPLE'S HEALTH PARTNERSHIP PCN
- PERSHORE PCN
- PIONEERS INTEGRATED PARTNERSHIP PCN
- QUINTON AND HARBORNE PCN
- SHARD END AND KITTS GREEN PCN
- SMALL HEATH PCN
- SMARTCARE CENTRAL PCN
- SOLIHULL HEALTHCARE PARTNERSHIP PCN
- SOLIHULL RURAL PCN
- SOLIHULL SOUTH CENTRAL PCN
- SOUTH BIRMINGHAM ALLIANCE PCN
- SOUTH WEST BIRMINGHAM PCN
- SUTTON GROUP PRACTICE PCN

- SWB I3 PCN
- SWB MODALITY PCN
- SWB URBAN HEALTH PCN
- WASHWOOD HEATH PCN
- WEOLEY AND RUBERY PCN
- WEST BIRMINGHAM PCN
- Other

49

Which is your Primary Care Network (PCN) in NHS Black Country ICB *

- ◯ BRIERLEY HILL PCN
- ◯ DUDLEY AND NETHERTON PCN
- ◯ HALESOWEN PCN
- ◯ HEALTH VISION PARTNERSHIP PCN
- ◯ KINGSWINFORD & WORDSLEY PCN
- ◯ OLDBURY & SMETHWICK PCN
- ◯ SEDGLEY, COSELEY & GORNAL PCN
- ◯ STOURBRIDGE, WOLLESCOTE & LYE PCN
- ◯ SWB CARITAS PCN
- ◯ SWB CENTRAL HEALTH PARTNERSHIPS PCN
- ◯ SWB CITRUS PCN
- ◯ SWB NEWCOMEN PCN
- ◯ SWB TOGETHER4HEALTHCARE PCN
- ◯ SWB YOUR HEALTH PARTNERSHIP PCN
- ◯ WALSALL EAST 1 PCN
- ◯ WALSALL EAST 2 PCN
- ◯ WALSALL NORTH PCN
- ◯ WALSALL SOUTH 1 PCN
- ◯ WALSALL SOUTH 2 PCN
- ◯ WALSALL WEST 1 PCN
- ◯ WALSALL WEST 2 PCN
- ◯ WOLVERHAMPTON NORTH NETWORK PCN
- ◯ WOLVERHAMPTON RWT PCN
- ◯ WOLVERHAMPTON SOUTH EAST PCN
- ◯ WOLVERHAMPTON TOTAL HEALTH PCN
- ◯ WOLVERHAMPTON UNITY EAST PCN
- ◯ WOLVERHAMPTON UNITY WEST PCN
- ◯ Other

50

Which is your Primary Care Network (PCN) in NHS Coventry and Warwickshire ICB *

- ARDEN PCN
- COVENTRY CENTRAL PCN
- COVENTRY NAVIGATION 1 PCN
- COVENTRY NORTH PCN
- CW UNITY PCN
- DENE AND STOUR VALLEYS PCN
- GO WEST PCN
- GP CONNECT PCN
- KENILWORTH AND WARWICK PCN
- LEAMINGTON NORTH PCN
- LEAMINGTON SOUTH PCN
- NORTH ARDEN PCN
- NUNEATON & BEDWORTH PCN
- RUGBY PCN
- SKYWARD PCN
- SOWE VALLEY PCN
- STRATFORD CENTRAL PCN
- WARWICKSHIRE EAST PCN
- WARWICKSHIRE RURAL PCN
- Other

51

Which is your Primary Care Network (PCN) in NHS Derby and Derbyshire ICB *

- ALFRETON RIPLEY CRICH & HEANOR PCN
- BELPER PCN
- CHESTERFIELD AND DRONFIELD PCN
- DERBY CITY NORTH PCN
- DERBY CITY SOUTH PCN
- DERBYSHIRE DALES PCN
- EREWASH PCN
- GLOSSOP PCN
- GREATER DERBY PCN
- HIGH PEAK & BUXTON PCN
- NORTH DERBYSHIRE PCN
- NORTH EAST DERBYSHIRE PCN
- NORTH HARDWICK AND BOLSOVER PCN
- OAKDALE PARK PCN
- PCCO PCN
- SOUTH DALES PCN
- SOUTH HARDWICK PCN
- SWADLINCOTE PCN
- Other

52

Which is your Primary Care Network (PCN) in NHS Herefordshire and Worcestershire ICB *

- EAST HEREFORDSHIRE PCN
- HEREFORDSHIRE HEREFORD CITY HMG PCN
- NORTH & WEST HEREFORDSHIRE PCN
- REDDITCH & BROMSGROVE & DISTRICT PCN
- REDDITCH & BROMSGROVE KINGFISHER PCN
- REDDITCH & BROMSGROVE NIGHTINGALES PCN
- SOUTH & WEST HEREFORDSHIRE PCN
- SOUTH WORCESTERSHIRE MALVERN TOWN PCN
- SOUTH WORCS DROITWITCH & OMBERSLEY PCN
- SOUTH WORCS PERSHORE & UPTON PCN
- SOUTH WORCS VALE OF EVESHAM HEALTH PCN
- SOUTH WORCS WORCESTER CITY GP PCN
- THE WBC (HEREFORD CITY) PCN
- WF NETWORK OF INDEPENDENT PRACTICES PCN
- WYRE FOREST HEALTH PARTNERSHIP PCN
- Other

53

Which is your Primary Care Network (PCN) in NHS Leicester, Leicestershire and Rutland ICB *

- ACROSS LEICESTER PCN
- BEACON (CHARNWOOD) PCN
- BELGRAVE & SPINNEY HILL PCN
- BOSWORTH PCN
- CARILLON PCN
- CITY CARE ALLIANCE PCN
- CROSS COUNTIES PCN
- FOSSEWAY PCN
- G3 PCN
- HINCKLEY CENTRAL PCN
- LEICESTER CENTRAL PCN
- LEICESTER CITY & UNIVERSITY PCN
- LEICESTER CITY SOUTH PCN
- MARKET HARBOROUGH & BOSWORTH PCN
- MELTON, SYSTON AND VALE PCN
- NORTH BLABY PCN
- NORTH WEST LEICESTERSHIRE PCN
- OADBY & WIGSTON PCN
- ORION PCN
- RUTLAND HEALTH PCN
- SALUTEM PCN
- SOAR VALLEY PCN
- SOUTH BLABY & LUTTERWORTH PCN
- THE LEICESTER FOXES PCN
- WATERMEAD PCN
- WILLOWS HEALTH PCN
- Other

54

Which is your Primary Care Network (PCN) in NHS Lincolnshire ICB *

- APEX PCN
- BOSTON PCN
- EAST LINDSEY PCN
- FIRST COASTAL PCN
- FOUR COUNTIES PCN
- GRANTHAM AND RURAL PCN
- IMP PCN
- K2 HEALTHCARE SLEAFORD PCN
- LINCOLN HEALTH PARTNERSHIP PCN
- MERIDIAN MEDICAL PCN
- SOUTH LINCOLN PCN
- SOUTH LINCOLNSHIRE RURAL PCN
- SPALDING PCN
- TRENT CARE PCN
- Other

55

Which is your Primary Care Network (PCN) in NHS Northamptonshire ICB *

- ◯ BLUE PCN
- ◯ BRACKLEY & TOWCESTER PCN
- ◯ DAVENTRY PCN
- ◯ EAST NORTHANTS PCN
- ◯ GRAND UNION PCN
- ◯ KETTERING & SOUTH WEST RURAL PCN
- ◯ MMWF PCN
- ◯ M-WEB PCN
- ◯ NORTHAMPTONSHIRE RURAL PCN
- ◯ PARKWOOD PCN
- ◯ RED KITE HEALTHCARE PCN
- ◯ ROCKINGHAM FOREST PCN
- ◯ ROYAL PARKS PCN
- ◯ THE ARC HUB PCN
- ◯ TRIANGLE PCN
- ◯ WELLINGBOROUGH & DISTRICT PCN
- ◯ Other

56

Which is your Primary Care Network (PCN) in NHS Nottingham and Nottinghamshire ICB *

- ARNOLD AND CALVERTON PCN
- ARROW HEALTH PCN
- ASHFIELD NORTH PCN
- ASHFIELD SOUTH PCN
- ASPIRE PCN
- BACHS PCN
- BESTWOOD AND SHERWOOD PCN
- BULWELL AND TOP VALLEY PCN
- BYRON PCN
- CITY SOUTH PCN
- CLIFTON & MEADOWS PCN
- LARWOOD & BAWTRY PCN
- MANSFIELD NORTH PCN
- NEWARK PCN
- NEWGATE MEDICAL GROUP PCN
- NOTTINGHAM CITY EAST PCN
- NOTTINGHAM WEST PCN
- RADFORD AND MARY POTTER PCN
- RALEIGH HEALTHCARE PCN
- RETFORD AND VILLAGES PCN
- ROSEWOOD PCN
- RUSHCLIFFE PCN
- SHERWOOD PCN
- SYNERGY HEALTH PCN
- UNITY (NOTTINGHAM) PCN
- Other

57

Which is your Primary Care Network (PCN) in NHS Shropshire, Telford and Wrekin ICB *

- NEWPORT AND CENTRAL PCN
- NORTH SHROPSHIRE PCN
- SE SHROPSHIRE PCN
- SHREWSBURY PCN
- SHROPSHIRE RURAL ALLIANCE PCN
- SOUTH EAST TELFORD PCN
- SW SHROPSHIRE PCN
- TELDOC PCN
- WREKIN PCN
- Other

58

Which is your Primary Care Network (PCN) in NHS Staffordshire and Stoke-on-Trent ICB *

- ABOUT BETTER CARE (ABC) PCN
- BURNTWOOD PCN
- CANNOCK NORTH PCN
- CANNOCK VILLAGES PCN
- EAST STAFFORDSHIRE PCN
- HANLEY, BUCKNALL & BENTILEE PCN
- HIPC (HOLISTIC PATIENT CENTRED CARE) PCN
- LEEK & BIDDULPH PCN
- LICHFIELD PCN
- MEIR PCN
- MERCIAN PCN
- MOORLANDS & RURAL PCN
- NEWCASTLE CENTRAL PCN
- NEWCASTLE NORTH PCN LIMITED
- NEWCASTLE SOUTH PCN
- RUGELEY & GREAT HAYWOOD PCN
- SEISDON PCN
- SHELTON & HANLEY PCN
- SOUTH STOKE CENTRAL PCN
- SOUTH STOKE WEST PCN
- STAFFORD CENTRAL PCN
- STAFFORD SOUTH PCN
- STAFFORD TOWN PCN
- STONE & ECCLESHALL PCN
- WHITFIELD PCN
- Other

North East and Yorkshire Primary Care Networks

59

Which is your Integrated Care Board (ICB) in North East and Yorkshire *

- ◯ NHS Humber and North Yorkshire ICB
- ◯ NHS North East and North Cumbria ICB
- ◯ NHS South Yorkshire ICB
- ◯ NHS West Yorkshire ICB

60

Which is your Primary Care Network (PCN) in NHS Humber and North Yorkshire ICB *

- ACCLAIM NHS PCN
- APOLLO PCN
- BEVERLEY PCN
- BRIDLINGTON PCN
- CYGNET EAST RIDING PCN
- FILEY AND SCARBOROUGH PCN
- FRESHNEY PELHAM NEL PCN
- HAMBLETON NORTH PCN
- HAMBLETON SOUTH PCN
- HARTHILL EAST RIDING PCN
- HASP PCN - HULL ASSOCIATION OF SIMILAR PRACTICES
- HAXBY HULL GROUP PCN
- HEART OF HARROGATE PCN
- HOLDERNESS PRIMARY CARE HOME PCN
- HULL MEDICAS PCN
- HULL MODALITY PARTNERSHIP PCN
- HULL SYMPHONIE PCN
- KNARESBOROUGH & RURAL PCN
- MARMOT PCN
- MERIDIAN HEALTH GROUP NEL PCN
- MOWBRAY SQUARE PCN
- NORTH LINCOLNSHIRE EAST PCN
- NORTH LINCOLNSHIRE NORTH PCN
- NORTH LINCOLNSHIRE SOUTH PCN
- NORTH LINCOLNSHIRE WEST PCN
- NORTH RIDING HEALTHY COMMUNITY PCN
- PANACEA PCN
- PRIORY MEDICAL GROUP YORK PCN
- RICHMONDSHIRE PCN

- ◯ RIPON & MASHAM PCN
- ◯ RIVER & WOLDS EAST RIDING PCN
- ◯ SCARBOROUGH CORE PCN
- ◯ SELBY TOWN PCN
- ◯ SLC MEDICAL GROUP PCN
- ◯ SOUTH HAMBLETON & RYEDALE PCN
- ◯ TADCASTER AND SELBY RURAL PCN
- ◯ VENN PCN
- ◯ WEST OUTER AND NORTH EAST YORK PCN
- ◯ WHITBY COAST & MOORS PCN
- ◯ YORK CITY CENTRE PCN
- ◯ YORK EAST PCN
- ◯ YORK MEDICAL GROUP PCN
- ◯ YORKSHIRE COAST & WOLDS PCN
- ◯ Other

61

Which is your Primary Care Network (PCN) in NHS North East and North Cumbria ICB *

- BILLINGHAM & NORTON PCN
- BIRTLEY AND CENTRAL GATESHEAD PCN
- BISHOP AUCKLAND PCN
- BLYTH PCN
- BYTES PCN
- CARLISLE HEALTHCARE PCN
- CARLISLE PCN
- CARLISLE RURAL PCN
- CENTRAL MIDDLESBROUGH PCN
- CHESTER LE STREET PCN
- CLAYPATH & UNIVERSITY MEDICAL GROUP PCN
- COALFIELDS PCN
- COCKERMOUTH & MARYPORT PCN
- COPELAND PCN
- CRAMLINGTON SEATON VALLEY PCN
- DARLINGTON PCN
- DERWENTSIDE PCN
- DURHAM COAST PCN
- DURHAM EAST PCN
- DURHAM WEST PCN
- EASINGTON CENTRAL PCN
- EAST CLEVELAND PCN
- EDEN (CUMBRIA) PCN
- ESTON PCN
- GATESHEAD CENTRAL SOUTH PCN
- GATESHEAD EAST PCN
- GATESHEAD INNER WEST PCN
- GATESHEAD OUTER WEST PCN
- GREATER MIDDLESBROUGH PCN

- HARTLEPOOL HEALTH PCN
- HARTLEPOOL PCN
- HOLGATE PCN
- JESMOND - LOWER GOSFORTH PCN
- KESWICK & SOLWAY PCN
- NEWCASTLE CENTRAL HEALTH PCN
- NEWCASTLE EAST PCN
- NEWCASTLE INNER WEST PCN
- NEWCASTLE OUTER WEST PCN
- NORTH EASINGTON PCN
- NORTH GOSFORTH PCN
- NORTH SHIELDS PCN
- NORTH STOCKTON PCN
- NORTH TYNESIDE NORTH WEST PCN
- NORTHUMBRIA PCN
- ONE LIFE HARTLEPOOL PCN
- REDCAR COASTAL PCN
- SEDGEFIELD 1 PCN
- SEDGEFIELD NORTH PCN
- SOUTH TYNESIDE EAST PCN
- SOUTH TYNESIDE SOUTH PCN
- SOUTH TYNESIDE WEST PCN
- STOCKTON PCN
- SUNDERLAND EAST PCN
- SUNDERLAND NORTH PCN
- SUNDERLAND WEST 1 PCN
- SUNDERLAND WEST 2 PCN
- TEESDALE PCN
- WALLSEND PCN
- WANSBECK PCN
- WASHINGTON PCN
- [illegible]

- WEAR VALLEY PCN
- WELL UP NORTH PCN
- WEST END FAMILY HEALTH PCN
- WEST NORTHUMBERLAND PCN
- WHITLEY BAY PCN
- WORKINGTON PCN
- Other

62

Which is your Primary Care Network (PCN) in NHS South Yorkshire ICB *

- 4 DONCASTER PCN
- BARNSLEY PCN
- CITY CENTRE AND UNIVERSITY SHU PCN
- DONCASTER CENTRAL PCN
- DONCASTER EAST PCN
- DONCASTER NORTH PCN
- DONCASTER NORTH WEST PCN
- DONCASTER SOUTH PCN
- FOUNDRY PCN
- GPA1 PCN
- HEALTH VILLAGE/DEARNE VALLEY PCN
- HEELEY PLUS PCN
- HILLSBOROUGH PCN
- MALTBY WICKERSLEY PCN
- NETWORK NORTH PCN
- PEAK EDGE PCN
- PORTER VALLEY PCN
- RAVEN PCN
- ROTHER VALLEY SOUTH PCN
- ROTHERHAM CENTRAL NORTH PCN
- SAPA 5 PCN
- SEVEN HILLS PCN
- STUDENT & CENTRAL SHEFFIELD PCN
- TOWNSHIPS 1 PCN
- TOWNSHIPS 2 PCN
- UOS STUDENT PCN
- UPPER DON VALLEY PCN
- WENTWORTH 1 PCN
- WEST 5 PCN

- [ ] Other

63

Which is your Primary Care Network (PCN) in NHS West Yorkshire ICB *

- 3 CENTRES PCN
- AFFINITY CARE PCN
- ARMLEY PCN
- BATLEY BIRSTALL PCN
- BD4+ PCN
- BEESTON PCN
- BRADFORD CITY 4 PCN
- BRADFORD CITY 5 PCN
- BRADFORD CITY 6 PCN
- BRADFORD NORTH WEST PCN
- BRAMLEY, WORTLEY & MIDDLETON PCN
- BRIGANTES PCN
- BURMANTOFTS, HAREHILLS & RICHMOND HILL PCN
- CALDER & RYBURN PCN
- CENTRAL HALIFAX PCN
- CENTRAL NORTH LEEDS PCN
- CHAPELTOWN PCN
- CROSSGATES PCN
- DEWSBURY & THORNHILL PCN
- FIVE LANE ENDS PCN
- FIVE TOWNS PCN
- GREENWOOD PCN
- HOLT PARK PCN
- LOWER VALLEY PCN
- LS25/LS26 PCN
- LSMP AND THE LIGHT PCN
- MIDDLETON AND HUNSLET PCN
- MODALITY (KEIGHLEY) PCN
- MORLEY PCN

North West Primary Care Networks

64

Which is your Integrated Care Board (ICB) in North West *

- NHS Cheshire and Merseyside ICB
- NHS Greater Manchester ICB
- NHS Lancashire and South Cumbria ICB

- THE VALLEYS HEALTH & SOCIAL CARE PCN
- TOLSON CARE PARTNERSHIP PCN
- TRINITY HEALTH GROUP PCN
- UPPER CALDER VALLEY PCN
- VIADUCT CARE PCN
- WACA PCN
- WAKEFIELD HEALTH ALLIANCE SOUTH PCN
- WAKEFIELD NORTH PCN
- WEST LEEDS PCN
- WEST WAKEFIELD PCN
- WETHERBY PCN
- WOODSLEY PCN
- YEADON PCN
- YORK ROAD PCN
- Other

65

Which is your Primary Care Network (PCN) in NHS Cheshire and Merseyside ICB *

- ANFIELD & EVERTON PCN
- BIRKENHEAD PCN
- CENTRAL & WEST WARRINGTON PCN
- CENTRAL LIVERPOOL PCN
- CHAW (CHELFORD, HANDFORTH, ALDERLEY EDGE, WILMSLOW) PCN
- CHESTER CENTRAL PCN
- CHESTER EAST PCN
- CHESTER SOUTH PCN
- CHILDWALL & WAVERTREE PCN
- CHOC (CONGLETON & HOLMES CHAPEL) PCN
- CREWE - GHR PCN
- EAGLE BRIDGE PCN
- EAST WARRINGTON PCN
- HEALTHIER SOUTH WIRRAL PCN
- HEALTHIER WEST WIRRAL PCN
- IGPC PCN
- KIRKBY PCN
- KNOWSLEY CENTRAL AND SOUTH PCN
- KNUTSFORD PCN
- LIVERPOOL FIRST PCN
- MACCLESFIELD PCN
- MIDDLEWOOD PCN
- MORETON AND MEOLS PCN
- NANTWICH & RURAL PCN
- NESTON & WILLASTON PCN
- NEWTON AND HAYDOCK PCN
- NORTH LIVERPOOL PCN
- NORTHWICH PCN
- ONE ELLESMERE PORT PCN

66

Which is your Primary Care Network (PCN) in NHS Greater Manchester ICB *

- ALTRINCHAM HEALTHCARE ALLIANCE PCN
- ARDWICK AND LONGSIGHT PCN
- ASHTON PCN
- BETTER HEALTH MCR PCN
- BOLTON CENTRAL PCN
- BRAMHALL AND CHEADLE HULME PCN LTD
- BREIGHTMET & LITTLE LEVER PCN
- BROUGHTON HEALTH ALLIANCE PCN
- BURY PCN
- CANALSIDE PCN
- CHEADLE NETWORK PCN
- CHEETHAM HILL & CRUMPSALL PCN
- CHORLEY ROADS NETWORK PCN
- CITY CENTRE & ANCOATS PCN
- CLAYTON BESWICK & OPENSHAW PCN
- DENTON PCN
- DIDSBURY CHORLTON PARK & BURNAGE PCN
- ECCLES & IRLAM PCN
- FARNWORTH & KEARSLEY PCN
- GORTON AND LEVENSHULME PCN
- H-BLACKLEY HARPURHEY & CHARLESTOWN PCN
- HEATONS GROUP NETWORK PCN
- HEYWOOD PCN
- HINDLEY PCN
- HORIZON PCN
- HORWICH NETWORK PCN
- HULME & CITY CENTRE SOUTH PCN
- HWL NETWORK PCN
- HYDE PCN

67

Which is your Primary Care Network (PCN) in NHS Lancashire and South Cumbria ICB *

- BARROW TOGETHER PCN
- BAY PCN
- BLACKBURN EAST PCN
- BLACKBURN NORTH PCN
- BLACKBURN WEST PCN
- BLACKPOOL CENTRAL PCN
- BLACKPOOL CENTRAL WEST PCN
- BLACKPOOL NORTH PCN
- BLACKPOOL SOUTH CENTRAL PCN
- BLACKPOOL SOUTH PCN
- BRIDGEDALE SOUTH RIBBLE PCN
- BURNLEY EAST PCN
- BURNLEY WEST PCN
- CARNFORTH AND MILNTHORPE PCN
- CHORLEY AND SOUTH RIBBLE NETWORK PCN
- CHORLEY CENTRAL PCN
- CHORLEY TOGETHER PCN
- DARWEN PCN
- FLEETWOOD PCN
- GRANGE AND LAKES PCN
- GREATER PRESTON PCN
- HYNDBURN CENTRAL PCN
- HYNDBURN RURAL PCN
- KENDAL PCN
- LANCASTER PCN
- LEYLAND PCN
- LYTHAM ST ANNES PCN
- MID FURNESS PCN
- MILLOM PCN

South East Primary Care Networks

68

Which is your Integrated Care Board (ICB) in South East *

- NHS Buckinghamshire, Oxfordshire and Berkshire West ICB
- NHS Frimley ICB
- NHS Hampshire and Isle of Wight ICB
- NHS Kent and Medway ICB
- NHS Surrey Heartlands ICB
- NHS Sussex ICB

- WYRE INTEGRATED NETWORK LTD PCN
- WYRE RURAL EXTENDED NEIGHBOURHOOD (WREN) PCN
- Other

69

Which is your Primary Care Network (PCN) in NHS Buckinghamshire, Oxfordshire and Berkshire West ICB *

- A34 WEST BERKSHIRE PCN
- ABINGDON AND DISTRICT PCN
- ABINGDON CENTRAL PCN
- ARC BUCKS PCN
- AYLESBURY CENTRAL PCN
- BANBURY ALLIANCE PCN
- BANBURY CROSS PCN
- BICESTER PCN
- CAVERSHAM PCN
- CENTRAL MAPLE PCN
- CHESHAM & LITTLE CHALFONT PCN
- CITY - EAST OXFORD PCN
- CITY - OX3+ PCN
- CYGNET PCN
- DASHWOOD PCN
- DIDCOT PCN
- EARLEY + PCN
- ELM PARK PCN
- EYNSHAM & WITNEY PCN
- HEALTHIER OXFORD CITY NETWORK PCN
- HENLEY SONNET PCN
- KENNET PCN
- KIDLINGTON, ISLIP, WOODSTOCK & YARNTON (KIWY) PCN
- MID CHILTERN PCN
- MODALITY WOKINGHAM PCN
- NEW READING PCN
- NORTH BUCKS PCN
- NORTH OXFORDSHIRE RURAL ALLIANCE (NORA) PCN
- OXFORD CENTRAL PCN

70

Which is your Primary Care Network (PCN) in NHS Frimley ICB *

- ALDERSHOT PCN
- ASCOT PCN
- BRACCAN PCN
- BRACKNELL AND DISTRICT PCN
- CENTRAL SLOUGH NETWORK PCN
- FARNBOROUGH PCN
- FARNHAM PCN
- FLEET PCN
- LOCC PCN
- MAIDENHEAD PCN
- SHAPE PCN
- SPINE PCN
- SURREY HEATH PCN
- THE HEALTH TRIANGLE PCN
- WINDSOR PCN
- YATELEY PCN
- Other

- WOKINGHAM NORTH PCN
- WOKINGHAM SOUTH PCN
- WOOSEHILL AND CROWTHORNE PCN
- Other

71

Which is your Primary Care Network (PCN) in NHS Hampshire and Isle of Wight ICB *

- A31 GROUP PCN
- ANDOVER PCN
- AVON VALLEY PCN
- BRUNEL PCN
- CAMROSE, GILLIES & HACKWOOD PARTNERSHIP PCN
- CENTRAL & WEST (IW) PCN
- CHANDLER'S FORD PCN
- COASTAL (WEST HAMPSHIRE) PCN
- COASTAL FAREHAM & GOSPORT PCN
- EAST HANTS PCN
- EASTLEIGH HEALTH PCN
- EASTLEIGH SOUTHERN PARISHES PCN
- FAREHAM & PORTCHESTER PCN
- GOSPORT CENTRAL PCN
- HAVANT AND WATERLOOVILLE PCN
- HAYLING ISLAND & EMSWORTH PCN
- ISLAND CITY PCN
- MEON HEALTH PCN
- MOSAIC HEALTHCARE PCN
- NEW FOREST PCN
- NORTH & EAST (IW) PCN
- PORTSDOWN PCN
- PORTSMOUTH NORTH PCN
- PORTSMOUTH SOUTH COAST PCN
- ROMSEY & NORTH BADDESLEY PCN
- RURAL WEST PCN
- SHAKESPEARE ROAD MEDICAL PRACTICE PCN
- SOUTH (IW) PCN
- SOUTHAMPTON BITTERNE PCN

72

Which is your Primary Care Network (PCN) in NHS Kent and Medway ICB *

- ABC PCN
- ASHFORD MEDICAL PARTNERSHIP PCN
- ASHFORD RURAL PCN
- ASPIRE MEDICAL PCN
- ATHENA PCN
- CANTERBURY NORTH PCN
- CANTERBURY SOUTH PCN
- CARE KENT PCN
- DARTFORD CENTRAL PCN
- DARTFORD MODEL PCN
- DEAL & SANDWICH PCN
- DOVER TOWN PCN
- FOLKESTONE HYTHE & RURAL PCN
- GARDEN CITY PCN
- GILLINGHAM SOUTH PCN
- GRAVESEND ALLIANCE PCN
- GRAVESEND CENTRAL PCN
- HERNE BAY PCN
- LMN PCN
- MAIDSTONE CENTRAL PCN
- MALLING PCN
- MARGATE PCN
- MEDWAY CENTRAL PCN
- MEDWAY PENINSULA PCN
- MEDWAY RAINHAM PCN
- MEDWAY SOUTH PCN
- MID KENT PCN
- MPA PCN
- RAMSGATE PCN

73

Which is your Primary Care Network (PCN) in NHS Surrey Heartlands ICB *

- BANSTEAD HEALTHCARE PCN
- CARE COLLABORATIVE (REDHILL) PCN
- CENTRAL AND NORTH GUILDFORD PCN
- COBHAM AND OXSHOTT PCN
- COCO PCN
- DORKING PCN
- EAST ELMBRIDGE PCN
- EAST WAVERLEY PCN
- EPSOM PCN
- GUILDFORD EAST PCN
- HEALTHY HORLEY PCN
- INTEGRATED CARE PARTNERSHIP PCN
- LEATHERHEAD PCN
- NORTH TANDRIDGE PCN
- REDHILL PHOENIX PCN
- SASSE NETWORK 1 PCN
- SASSE NETWORK 2 PCN
- SASSE NETWORK 3 PCN
- SOUTH TANDRIDGE PCN
- WALTON PRACTICES CONFEDERATION PCN
- WB PCN
- WEST OF WAVERLEY PCN
- WHAM PCN
- WOKING WISE 1 PCN
- WOKING WISE 2 PCN
- WOKING WISE 3 PCN
- Other

74

Which is your Primary Care Network (PCN) in NHS Sussex ICB *

- ALPS GROUP PCN
- ANGMERING COPPICE FITZALAN (ACF) PCN
- ARUN INTEGRATED CARE (AIC) PCN LTD
- BEXHILL PCN
- BOGNOR COASTAL ALLIANCE PCN
- BURGESS HILL & VILLAGES PCN
- CENTRAL WORTHING PRACTICES PCN
- CHANCTONBURY PCN
- CHICHESTER ALLIANCE OF MEDICAL PRACTICES PCN
- CISSBURY INTEGRATED CARE PCN
- COASTAL AND SOUTH DOWNS PCN
- CRAWLEY CARE COLLABORATIVE PCN
- DEAN'S AND CENTRAL BRIGHTON PCN
- EAST & CENTRAL BRIGHTON PCN
- EAST GRINSTEAD PCN
- EASTBOURNE EAST PCN
- FOUNDRY HEALTHCARE LEWES PCN
- GOLDSTONE PCN
- GREATER WEALDEN PCN
- HAILSHAM PCN
- HASTINGS & ST LEONARDS PCN
- HAYWARDS HEATH CENTRAL PCN
- HAYWARDS HEATH VILLAGES PCN
- HEALTHY CRAWLEY PCN
- HIGH WEALD PCN
- HORSHAM CENTRAL PCN
- HORSHAM COLLABORATIVE PCN
- LANCING AND SOMPTING PCN
- NORTH & CENTRAL BRIGHTON PCN

South West Primary Care Networks

75

Which is your Integrated Care Board (ICB) in South West *

- NHS Bath and North East Somerset, Swindon and Wiltshire ICB
- NHS Bristol, North Somerset and South Gloucestershire ICB
- NHS Cornwall and The Isles of Scilly ICB
- NHS Devon ICB
- NHS Dorset ICB
- NHS Gloucestershire ICB
- NHS Somerset ICB

76

Which is your Primary Care Network (PCN) in NHS Bath and North East Somerset, Swindon and Wiltshire ICB *

- BATH INDEPENDENTS PCN
- BRADFORD ON AVON & MELKSHAM PCN
- BRUNEL HEALTH GROUP PCN1
- BRUNEL HEALTH GROUP PCN2
- BRUNEL HEALTH GROUP PCN3
- BRUNEL HEALTH GROUP PCN4
- BRUNEL HEALTH GROUP PCN5
- BRUNEL HEALTH GROUP PCN6
- CALNE PCN
- CHIPPENHAM, CORSHAM & BOX PCN
- DEVIZES PCN
- EAST KENNET PCN
- HAWTHORN AND MERCHISTON PCN
- HEART OF BATH PCN
- KEYNSHAM PCN
- MINERVA HEALTH GROUP PCN
- NORTH WILTS BORDER PCN
- SALISBURY PLAIN PCN
- SARUM CATHEDRAL
- SARUM NORTH PCN
- SARUM TRINITY PCN
- SARUM WEST PCN
- SPARCELLS PCN
- THREE VALLEYS HEALTH PCN
- TROWBRIDGE PCN
- UNITY MEDICAL GROUP PCN
- WESTBURY & WARMINSTER PCN
- WYVERN HEALTH PARTNERSHIP PCN
- Other

77

Which is your Primary Care Network (PCN) in NHS Bristol, North Somerset and South Gloucestershire ICB *

- ◯ 4PCN (BNSSG) PCN
- ◯ AFFINITY (BNSSG) PCN
- ◯ BRIDGE VIEW PCN
- ◯ BRISTOL INNER CITY PCN
- ◯ CONCORD MENDIP PCN
- ◯ CONNEXUS PCN
- ◯ FABB (FISHPONDS, AIR BALLOON & BEECHWOOD) PCN
- ◯ FOSS (FIRECLAY & OLD SCHOOL SURGERY) PCN
- ◯ GORDANO VALLEY PCN
- ◯ HEALTHWEST PCN
- ◯ MENDIP VALE PCN
- ◯ NETWORK 4 (BNSSG) PCN
- ◯ NORTHERN ARC PCN
- ◯ PHOENIX (BNSSG) PCN
- ◯ PIER HEALTH PCN
- ◯ SEVERNVALE PCN
- ◯ STOKES PCN
- ◯ SWIFT PCN
- ◯ TYNTESFIELD PCN
- ◯ YATE & FRAMPTON PCN
- ◯ Other

78

Which is your Primary Care Network (PCN) in NHS Cornwall and The Isles of Scilly ICB *

- ARBENNEK HEALTH PCN
- COASTAL (KERNOW) PCN
- EAST CORNWALL PCN
- FALMOUTH AND PENRYN PCN
- ISLES OF SCILLY & SOUTH KERRIER PCN
- LAUNCESTON AND TAMAR VALLEY PCN
- NORTH CORNWALL COAST PCN
- NORTH KERRIER EAST PCN
- NORTH KERRIER WEST PCN
- PENWITH PCN
- ST AUSTELL HEALTHCARE PCN
- THREE HARBOURS AND BOSVENA PCN
- TRURO PCN
- WATERGATE PCN
- Other

79

Which is your Primary Care Network (PCN) in NHS Devon ICB *

- BARNSTAPLE ALLIANCE PCN
- BAYWIDE PCN
- BEACON MEDICAL GROUP PCN
- BRIXHAM AND PAIGNTON PCN
- COAST AND COUNTRY PCN
- CULM VALLEY PCN
- DRAKE MEDICAL ALLIANCE PCN
- EXETER CITY PCN
- EXETER WEST PCN
- HONITON/OTTERY/SID VALLEY (HOSMS) PCN
- LIVEWELL MAYFLOWER PCN
- MEWSTONE PCN
- MID DEVON HEALTHCARE PCN
- NEWTON WEST PCN
- NEXUS (DEVON) PCN
- NORTH DARTMOOR PCN
- NORTH DEVON COASTAL PCN
- OUTER EXETER PCN
- PATHFIELDS MEDICAL GROUP PCN
- SOUND PCN
- SOUTH DARTMOOR AND TOTNES PCN
- SOUTH HAMS PCN
- TASC PCN
- TEMPLER CARE NETWORK PCN
- THE COASTAL NETWORK PCN
- TIVERTON PCN
- TORQUAY PCN
- TORRIDGE PCN
- WATERSIDE HEALTH NETWORK PCN

80

Which is your Primary Care Network (PCN) in NHS Dorset ICB *

- BLANDFORD PCN
- BOURNEMOUTH EAST COLLABORATIVE PCN
- CENTRAL BOURNEMOUTH PCN
- CHRISTCHURCH PCN
- CRANE VALLEY PCN
- JURASSIC COAST PCN
- MID DORSET PCN
- NORTH BOURNEMOUTH PCN
- POOLE BAY & BOURNEMOUTH PCN
- POOLE CENTRAL PCN
- POOLE NORTH PCN
- PURBECK PCN
- SHERBORNE AREA PCN
- SHORE MEDICAL PCN
- SOUTH COASTAL MEDICAL GROUP PCN
- THE VALE PCN
- WEYMOUTH & PORTLAND PCN
- WIMBORNE & FERNDOWN PCN
- Other

81

Which is your Primary Care Network (PCN) in NHS Gloucestershire ICB *

- ◯ ASPEN PCN
- ◯ BERKELEY VALE PCN
- ◯ CHELTENHAM CENTRAL PCN
- ◯ CHELTENHAM PERIPHERAL PCN
- ◯ FOREST OF DEAN PCN
- ◯ GLOUCESTER INNER CITY PCN
- ◯ HADWEN QUEDGELEY PCN
- ◯ NORTH & SOUTH GLOUCESTER (NSG) PCN
- ◯ NORTH COTSWOLDS PCN
- ◯ ROSEBANK PCN
- ◯ SEVERN HEALTH PCN
- ◯ SOUTH COTSWOLDS PCN
- ◯ ST PAUL'S PCN
- ◯ STROUD COTSWOLD PCN
- ◯ TWNS PCN
- ◯ Other

82

Which is your Primary Care Network (PCN) in NHS Somerset ICB *

- BRIDGWATER PCN
- CHARD, ILMINSTER & LANGPORT (CLICK) PCN
- FROME PCN
- MENDIP PCN
- NORTH SEDGEMOOR PCN
- SOUTH SOMERSET EAST - RPN PCN
- SOUTH SOMERSET WEST PCN
- TAUNTON CENTRAL PCN
- TAUNTON DEANE WEST PCN
- TONE VALLEY PCN
- WEST MENDIP PCN
- WEST SOMERSET PCN
- YEOVIL PCN
- Other

83

Thank you for taking the time to complete this survey. Your responses will help identify the current uptake and utilisation of CES guidance in the Primary Care setting.

By pressing this button, you are submitting the survey. This survey is anonymous; therefore, once submitted, you will not be able to withdraw your responses.

Anonymised and aggregated data will be grouped into Integrated Care Board level data and shared on the Open Science Framework. If you wish to opt out of your data being shared please check the box below.

Our team would be extremely grateful if you could assist further by sharing the link to this survey with all eligible colleagues (i.e., have a job role in the **Primary Care setting** and see **people with back pain**)

○ Opt out of data sharing

84

Thank you for your interest in this survey. Unfortunately, you do not meet the eligibility criteria for this study. However, further research on this topic is planned in the near future that you may be eligible for.

Anonymised and aggregated data will be grouped into Integrated Care Board level data and shared on the Open Science Framework. If you wish to opt out of your data being shared please check the box below.

Our team would be extremely grateful if you could assist further by sharing the link to this survey with all eligible colleagues (i.e., have a job role in the **Primary Care setting** and see **people with back pain**)

◯ Opt out of data sharing

85

Thank you for taking the time to complete this survey. Most survey questions are related to cauda equina syndrome guidelines. As you have stated you are not aware of any guidelines subsequent questions will not be relevant. However, further research on this topic is planned in the near future that may be of interest to you.

Anonymised and aggregated data will be grouped into Integrated Care Board level data and shared on the Open Science Framework. If you wish to opt out of your data being shared please check the box below.

Our team would be extremely grateful if you could assist further by sharing the link to this survey with all eligible colleagues (i.e., have a job role in the **Primary Care setting** and see **people with back pain**)

◯ Opt out of data sharing

86

Thank you for your interest in this survey. Unfortunately, you do not meet the eligibility criteria for the study. The study is investigating the utilisation of guidance for patients suspected of having cauda equina syndrome in **England only**.

This content is neither created nor endorsed by Microsoft. The data you submit will be sent to the form owner.

Microsoft Forms
